# A Modular In Vitro Platform for the Production of Terpenes and Polyketides from CO_2_

**DOI:** 10.1002/anie.202102333

**Published:** 2021-06-03

**Authors:** Srividhya Sundaram, Christoph Diehl, Niña Socorro Cortina, Jan Bamberger, Nicole Paczia, Tobias J. Erb

**Affiliations:** ^1^ Department of Biochemistry and Synthetic Metabolism Max Planck Institute for Terrestrial Microbiology Karl-von-Frisch-Strasse 10 35043 Marburg Germany; ^2^ Equipment Center for Mass Spectrometry and Elemental Analysis Department of Chemistry Philipps-Universität Marburg Hans-Meerwein-Strasse 4 35043 Marburg Germany; ^3^ Core Facility for Metabolomics and Small Molecule Mass Spectrometry Max Planck Institute for Terrestrial Microbiology Karl-von-Frisch-Strasse 10 35043 Marburg Germany

**Keywords:** biocatalysis, CO_2_ fixation, in vitro biochemistry, reaction cascades, terpenes

## Abstract

A long‐term goal in realizing a sustainable biocatalysis and organic synthesis is the direct use of the greenhouse gas CO_2_ as feedstock for the production of bulk and fine chemicals, such as pharmaceuticals, fragrances and food additives. Here we developed a modular in vitro platform for the continuous conversion of CO_2_ into complex multi‐carbon compounds, such as monoterpenes (C_10_), sesquiterpenes (C_15_) and polyketides. Combining natural and synthetic metabolic pathway modules, we established a route from CO_2_ into the key intermediates acetyl‐ and malonyl‐CoA, which can be subsequently diversified through the action of different terpene and polyketide synthases. Our proof‐of‐principle study demonstrates the simultaneous operation of different metabolic modules comprising of up to 29 enzymes in one pot, which paves the way for developing and optimizing synthesis routes for the generation of complex CO_2_‐based chemicals in the future.

## Graphical abstract

Coupling of a synthetic CO_2_‐fixing cycle and a natural glyoxylate assimilation cycle with different biosynthetic pathways enables the sustainable production of terpenes and polyketides. Demonstration of this modular, multi‐enzyme in vitro platform sets the stage for a diversified synthesis of pharmaceuticals and commodity chemicals directly from CO_2_.

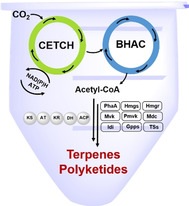

Cell‐free synthetic biology involves the in vitro assembly of multiple purified and semi‐purified enzymes into metabolic cascades to generate natural and new‐to‐nature high‐value chemicals. The in vitro reconstitution of metabolic pathways allows the convenient manipulation and optimization of reaction conditions, enzyme concentrations, cofactor supply, energy flux and yield, which are difficult to control in living microorganisms.^[1]^ Glucose is frequently used as an inexpensive feedstock for these complex systems. Over the recent years, the use of other carbon sources, such as sucrose, cellulose, glycerol, xylose and starch, was demonstrated.^[2]^ However, the direct conversion of atmospheric CO_2_ or other C_1_ precursors into value‐added compounds has proven a major challenge for in vitro systems.

To address this challenge, the synthetic crotonyl‐coenzyme (CoA)/ethylmalonyl‐CoA/hydroxybutyryl‐CoA cycle (CETCH) was developed. This in vitro pathway combines 18 enzymes from nine different organisms to generate the C_2_ compound glyoxylate from CO_2_ at a rate of 5 nmoles per minute per mg protein.^[3]^ Very recently, CETCH was successfully coupled to photosynthetic membranes for the light‐driven conversion of CO_2_ into glycolate.^[4]^ However, a successful coupling of CETCH to downstream anabolic pathway modules that would allow to extend its product spectrum beyond glyoxylate or glycolate is still lacking. One interesting set of target molecules are natural products, in particular terpenes and polyketides, which are used as flavors, pharmaceuticals, biofuels and commodity chemicals. These complex compounds are synthesized in vivo from the simple C_2_ building blocks like acetyl‐CoA by individual enzymes (terpene synthases) or multi‐enzyme complexes (polyketide synthases, PKSs) respectively.^[5]^

Here we developed a multi‐modular in vitro platform to access different terpenes and polyketides directly from CO_2_. To that end, we first coupled the synthetic CETCH with a natural glyoxylate assimilation module to convert CO_2_ into acetyl‐CoA. We further demonstrate how acetyl‐CoA can be diversified into an array of terpenes and polyketides through downstream processing by different terpene and PKS biosynthetic modules. Overall, this proof‐of‐principle study might pave the way towards realizing modular, multi‐enzyme reaction cascades for the sustainable synthesis of complex chemicals from simple C_1_ building blocks, such as CO_2_, in the future.

To capture CO_2_ into glyoxylate, we first established CETCH and determined its productivity in our experimental setup. To that end, we run CETCH version 5.4 (Supplementary Information) and quantified glycolate production by adding glyoxylate reductase to the CETCH core cycle. Starting from 100 μm propionyl‐CoA, CETCH produced approximately 730 μm glycolate within 3 hours under the chosen conditions (Figure 1 A).

**Figure 1:**
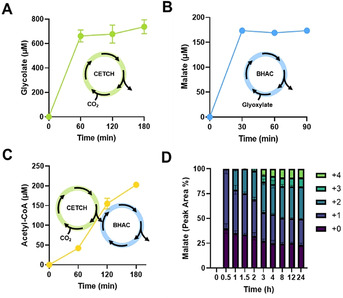
Coupling of CETCH with the BHAC for acetyl‐CoA production. A) Glycolate production by CETCH from 100 μm propionyl‐CoA. Glyoxylate reductase was used to convert the primary product glyoxylate to glycolate. B) Malate production by BHAC from 500 μm glyoxylate. Malate dehydrogenase was used to convert oxaloacetate to malate. C) Coupling of CETCH with the BHAC to produce acetyl‐CoA (see Scheme 1) from 100 μm propionyl‐CoA. D) Fractional labeling of malate from CETCH–BHAC coupling and Mdh using ^13^C‐labeled bicarbonate and 100 μm propionyl‐CoA. The ^13^C is incorporated as CO_2_ by the Ccr as shown in Scheme 1. +0, +1, +2, +3, +4 indicates the number of carbons of the malate (C_4_) derived from ^13^CO_2_ incorporation. The reactions were performed in triplicates and the mean ± S.D. are plotted.

Next, we aimed at establishing a coupling module for the further conversion of glyoxylate into acetyl‐CoA, which would allow to couple CETCH with downstream terpene/polyketide‐producing pathways. We sought to employ the β‐hydroxyaspartate cycle (BHAC), a pathway used by marine proteobacteria for glyoxylate assimilation.^[6]^ The BHAC converts two molecules of glyoxylate into oxaloacetate via four enzymes, requiring only one molecule of NADH and one amino group that is constantly recycled during the process, making the BHAC the most efficient reaction sequence for the conversion of C_2_ molecules into C_4_ compounds described to date. Oxaloacetate can then be further converted into acetyl‐CoA via malate dehydrogenase, malate thiokinase and malyl‐CoA lyase (Scheme 1). We reconstituted the BHAC in vitro using N‐terminal His‐tagged proteins produced in *Escherichia coli* (Table S5). To optimize BHAC productivity, we tested different concentrations of transaminase BhcA and co‐substrate glycine, using malate dehydrogenase as readout (see Supplementary Information). However, starting from 500 μm glyoxylate, malate yields were comparable between the different conditions tested (approx. 70 %; Figure S1A), indicating that the BHAC was operating robustly in vitro (Figure 1 B). Next, we coupled the BHAC with CETCH. When we added the enzymes of the BHAC after 60 min to the CETCH assay, CETCH plus BHAC yielded approximately 200 μm acetyl‐CoA, corresponding to a conversion of glyoxylate into acetyl‐CoA at 30 % yield (Figure S1B).

**Scheme 1:**
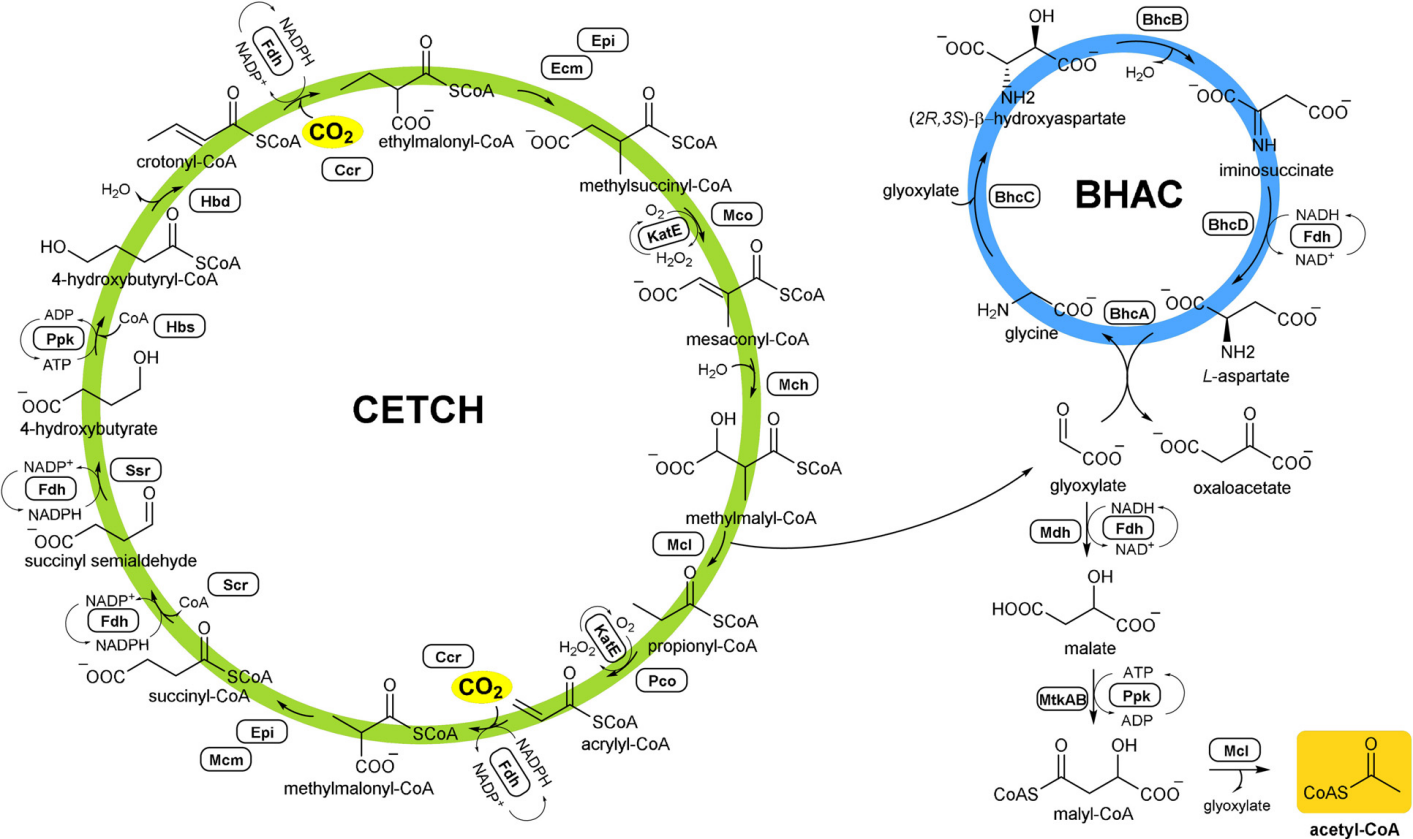
Coupling of CETCH–BHAC modules for acetyl‐CoA formation.

Notably, we achieved similar acetyl‐CoA yields when we coupled the CETCH with BHAC directly from the beginning (Figure 1 C and S1B), indicating that the 18 enzymes of the CETCH and BHAC can be operated simultaneously in one pot. CO_2_ fixation was also confirmed by isotopic labeling as before.^[3]^ Using ^13^C‐labeled bicarbonate and ^13^C‐formate (released as ^13^CO_2_ during cofactor recycling), fully labeled malate was observed after two hours, proving that CETCH turned multiple times (Figure 1 D). Considering that already single ^13^C‐labeled malate is stoichiometrically exclusively derived from fixed CO_2_ (Figure S2), these experiments demonstrated that CO_2_ can be continuously converted into acetyl‐CoA by directly coupling CETCH and BHAC.

The last step in the CETCH–BHAC cascade, the cleavage of malyl‐CoA into glyoxylate and acetyl‐CoA by malyl‐CoA lyase, is reversible with a Δ*G* ^0′^ of −3±5.8 kJ mol^−1^.^[3]^ To test whether this reaction runs into an equilibrium, we determined malyl‐CoA concentrations after 90 min. Much to our surprise, the concentration of malyl‐CoA was below 1 μm (Figure S3A), indicating that this compound is specifically degraded over time in the reaction mixture. Indeed, when we incubated both acetyl‐CoA and malyl‐CoA in the assay matrix in the absence and presence of all CETCH and BHAC enzymes (with exception of malyl‐CoA lyase Mcl), acetyl‐CoA appeared stable, while malyl‐CoA was consumed by one or more of the enzymes (Figure S3B,C). Thus, when coupling CETCH and BHAC, the last reaction in the cascade reaches an equilibrium between acetyl‐CoA plus glyoxylate and malyl‐CoA. Malyl‐CoA will be degraded over time, thereby limiting total yield, unless the flux is further pulled into downstream reactions that consume acetyl‐CoA (see below).

For the further conversion of acetyl‐CoA into terpenes, we aimed at coupling the 18 enzymes of the CETCH–BHAC cascade with different terpene biosynthetic modules, comprised of the nine enzymes of the mevalonate biosynthetic pathway and various terpene synthases (Figure 2 A, Table S5). We established five different terpene biosynthetic modules by prototyping the production of monoterpenes (C_10_) limonene (**1**), sabinene (**2**) and α‐pinene (**3**), as well as sesquiterpenes (C_15_) α‐bisabolene (**4**) and β‐farnesene (**5**) from acetyl‐CoA in the CETCH–BHAC assay matrix (Table S5). To constantly supply cofactors, we employed the regeneration systems used in CETCH. To regenerate the NAD(P)H pool, we used an engineered formate dehydrogenase (Fdh) that accepts both NADPH and NADH;^[7]^ to maintain the ATP pool, we used a polyphosphate transferase system.^[8]^ The extraction of terpenes was optimized by testing different solvents (Figure S4,B). Production of **1**–**5** from acetyl‐CoA was validated with authentic standards and further optimized by testing different terpene synthase concentrations (Figure S5).

**Figure 2:**
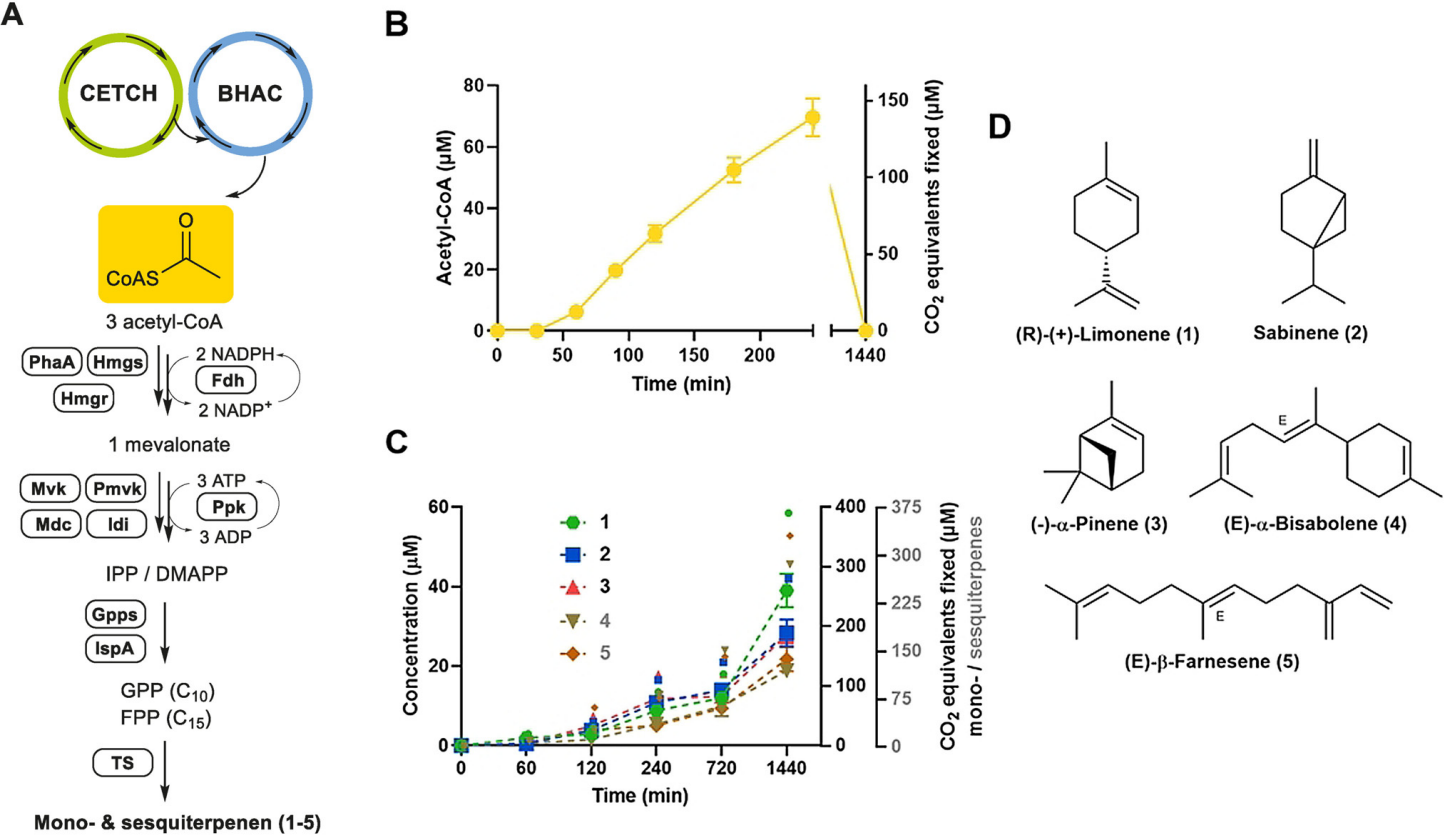
Coupling of CETCH–BHAC cycles for terpene biosynthesis. A) General scheme of the mevalonate pathway. The cofactors (NADPH and ATP) are fluxed from the CETCH/BHAC cycles and are constantly recycled. IPP: isopentenyl pyrophosphate; DMAPP: dimethylallyl pyrophosphate; TS: terpene synthase. B) Formation of acetyl‐CoA from CETCH over 24 h. The reaction is started with 100 μm propionyl‐CoA and analyzed as described in the methods. C) Time course of terpenes production from 100 μm propionyl‐CoA. Colored labels correspond to the amount of CO_2_ fixed over time. The analytes are measured by GC–MS as described in the methods. The reactions were performed in triplicates and the mean ± S.D. are plotted. D) Structures of the mono‐ and sesquiterpenes produced.

When we pre‐produced acetyl‐CoA with the CETCH–BHAC cascade for 4 h before adding the different terpene biosynthetic modules, monoterpenes **1**–**3** and sesquiterpenes **4**–**5** were produced at concentrations of approximately 10 and 5 μm, respectively (Figure S6B). However, when operating the CETCH–BHAC cascade with the different terpene biosynthetic modules simultaneously in one pot, product yields were increased three‐ to four‐fold (Figure S6C). The CETCH–BHAC cascade alone produced about 70 μm acetyl‐CoA within 4 h (Figure 2 B). We obtained monoterpenes **1**–**3** between concentrations of 30 and 40 μm, and sesquiterpenes **4** and **5** at 20 μm (Figure 2 C,D and Table 1), supporting the hypothesis that the direct downstream conversion of acetyl‐CoA is crucial for improving product yield. LC–MS analysis of the reaction mixtures confirmed the presence of different mevalonate pathway intermediates (Figure S7B), but only trace amounts of residual acetyl‐CoA (Figure S7C), indicating that acetyl‐CoA is efficiently fed into the different downstream terpene biosynthetic modules. As a positive control, the production of **1**–**5** was also validated from 0.5 mm acetyl‐CoA (Figure S7D).

**Table 1:** Net productivity of terpenes from 100 μm propionyl‐CoA in 24 h. Yield was determined by GC–MS using authentic standards (Figure S8). Data represent *n*=3 ± S.D. The assay contained 4.6 mg mL^−1^ of total enzymes.

Compounds	Yield [μm]	CO_2_ fixed [μm]	Productivity [mg L^−1^ h^−1^]
**1**	39±4	390	0.22
**2**	28±3	283	0.16
**3**	27±2	279	0.16
**4**	19±2	283	0.19
**5**	22±3	327	0.22

To test whether the CETCH–BHAC cascade would also fuel polyketide biosynthesis, we attempted to couple it with the iterative PKS C‐1027 (PKS_SgcE_).^[9]^ PKS_SgcE_ has been reported to form 1,3,5,7,9,11,13‐pentadecaheptaene (PDH, **7**), an all‐*trans* polyene, which upon chemical hydrogenation leads to pentadecane (PD), a prime component of diesel fuel. PKS_SgcE_ uses one acetyl‐CoA, eight malonyl‐CoA and seven NADPH to generate a nine‐membered enediyne precursor (Figure 3 A). The PKS undergoes eight iterative cycles, during which the dehydratase (DH) domain remains inactive in the last two cycles and the ketoreductase (KR) domain in the ultimate cycle.

**Figure 3:**
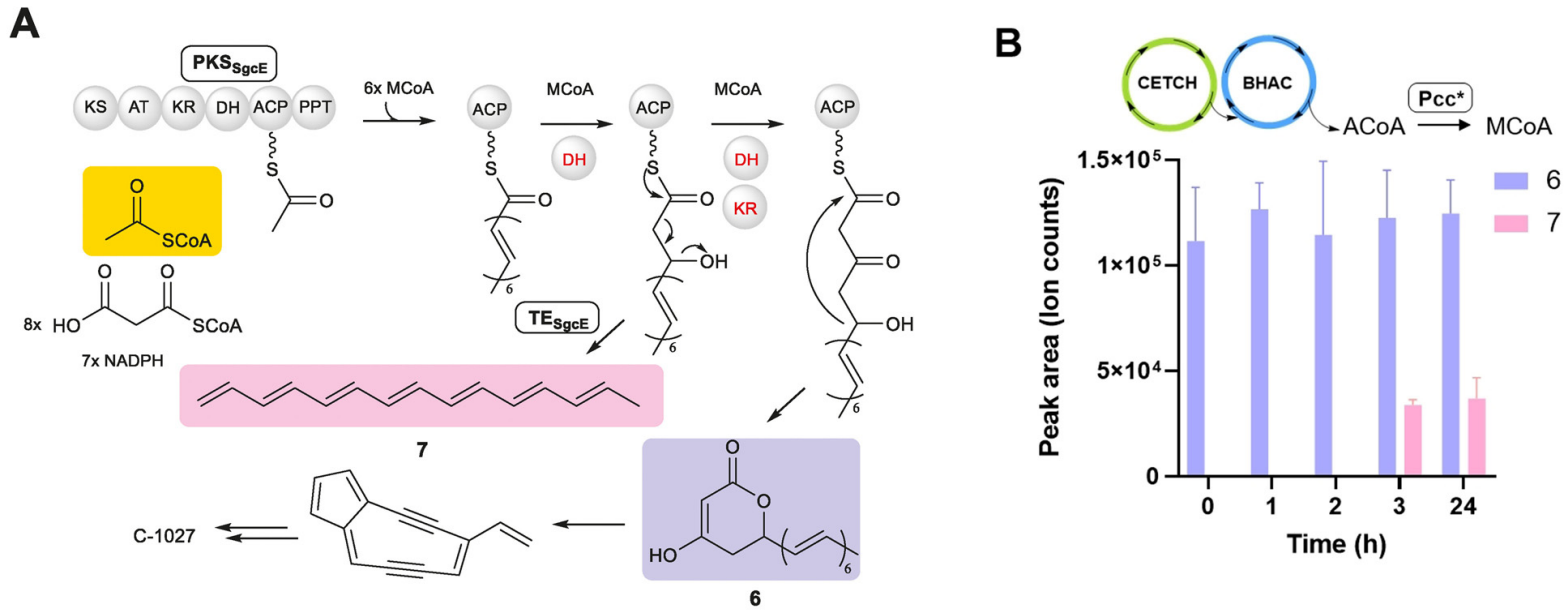
Coupling of CETCH–BHAC cycles for pentadecaheptaene biosynthesis. A) Proposed catalysis by PKS_SgcE_ and TE_SgcE_ in the biosynthesis of enediyne antibiotic C‐1027. KS: ketosynthase; AT: acyltransferase; KR: ketoreductase; DH: dehydratase; ACP: acyl carrier protein; PPT: phosphopantethenyl transferase. B) Formation of **7** via CETCH–BHAC from 100 μm propionyl‐CoA at different time points. The production of **7** stalls at 3 h and remains the same until 24 h. All the assays were performed in triplicates and the mean ± S.D. are plotted.

Products are released from PKS_SgcE_ either via spontaneous lactonisation yielding **6,** or through hydrolysis by the type II‐standalone thioesterase[^9^, ^10^] TE_SgcE_, yielding **7**. A recent study reported that production of **7** depends on the ratio of PKS_SgcE_:TE_SgcE_.^[11]^ We protoyped the in vitro production of **6** and **7** by mixing various concentrations of PKS_SgcE_ and TE_SgcE_ with acetyl‐CoA, malonyl‐CoA and NADPH at 30 °C. Production of **6** was directly confirmed from the reaction mixture with high‐resolution LCMS (*m*/*z*[M+H]^+^=285.1485). Production of **7** was confirmed in ethyl acetate extracts of the reaction mixture by UV/Vis spectroscopy (absorption maxima at 336, 355, 373, 395 nm) and high‐resolution LCMS (*m*/*z*[M+H]^+^=199.1476, Figure S10A). The amount of **7** increased with increasing TE_SgcE_ concentrations with a maximum production at 2.5 μm PKS_SgcE_ and 40 μm TE_SgcE_ (Figure S10B). Neither **6** nor **7** were detected when we tested PKS_SgcE_ mutant C171A in which the KS was inactivated (Figure S10C), demonstrating successful reconstitution of PKS_SgcE_ in vitro.

Note that CETCH features methylmalonyl‐CoA and ethylmalonyl‐CoA as intermediates, which serve as extender units in the biosynthesis of several polyketides and might pose a problem when directly coupling CETCH with PKS. To study whether PKS_SgcE_ would accept methyl‐ and ethylmalonyl‐CoA besides malonyl‐CoA we tested these compounds separately and in combination with purified PKS_SgcE_ and analyzed the reaction mixture for the production methyl‐ (**8**) and ethyl‐substituted (**9**) heptaenes with high‐resolution LCMS. Indeed, **8** and **9** were produced in a TE‐dependent fashion when the corresponding precursors were available (Figure S10D, I, II, III). However, PKS_SgcE_ clearly preferred production of **7** (Figure S10D IV, V, VI), suggesting that operating CETCH and PKS_SgcE_ simultaneously does not pose a challenge, as long as a sufficient pool of malonyl‐CoA is present.

Having this information at hand, we finally directly coupled the CETCH–BHAC cascade with 2.5 μm PKS_SgcE_ and 40 μm TE_SgcE_ and added 2 μm propionyl‐CoA carboxylase variant D407I (Pcc*) that shows 10 % activity with acetyl‐CoA to provide the extender unit malonyl‐CoA from acetyl‐CoA. In the coupled system, **6** was produced in relatively high amounts independent of TE_SgcE_, while TE_SgcE_‐dependent production of **7** reached a maximum around 3 h, demonstrating the successful biosynthesis of complex polyketides with our coupled system (Figure 3 B).

In conclusion, we show that more complex molecules such as terpenes and polyketides can be built exclusively from CO_2_ combining the synthetic CO_2_‐fixing CETCH with different biosynthetic modules. Although cofactor regeneration in our modular platform was based on formate, we note that this C_1_ compound can be regenerated electrochemically and/or enzymatically from CO_2_ and thus provide a carbon neutral energy (and carbon) source.^[12]^ Moreover, CETCH was recently coupled with chloroplast extracts.^[4]^ Energizing our modular platform with photosynthetic membranes could make our multi‐enzyme system completely independent of chemical energy in the future.

The net productivity of terpenes from CO_2_ reached with our modular platform is currently approximately 0.2 mg L^−1^ h^−1^. This is lower compared to glucose‐based cell‐free protein synthesis (CFPS) and other in vitro production systems, which range between 2 and 100 mg L^−1^ h^−1^.^[13]^ In vivo, **5** has been produced up to 2 g L^−1^ h^−1^ in *S. cerevisiae* by combining an artificial acetyl‐coA biosynthetic pathway with the NADH‐preferring Hmgr.^[14]^ The maximum titers reported for **2** and **3** are 15 mg L^−1^ h^−1^, 100 mg L^−1^ h^−1^ and 1.2 mg L^−1^ h^−1^ in *E. coli* under fed‐batch or shake flask fermentations,^[15]^ whereas the production of **4** reached 13 mg L^−1^ h^−1^ in both *E. coli* and *S. cerevisiae*.^[16]^ However, it should be noted that these production rates are based on the direct supply of multi‐carbon compounds and were achieved only after rigorous optimization of the different pathways both in vivo and in vitro.

It is conceivable that the productivity of our in vitro system can also be enhanced further by optimizing enzyme concentrations, activity and stability (e.g., through immobilization or the use of thermostable enzyme variants). For example, the mevalonate module itself has already been demonstrated to be self‐sustaining over a long period of time (approx. 7 days). Using modelling approaches or computer‐aided design‐build‐test cycles focusing on identifying optimal enzyme stoichiometries, intermediate concentrations and critical reaction parameters could further increase production rates of our in vitro system. Moreover, the product portfolio of our platform can be further expanded. Using natural, engineered and chimeric terpene synthases or PKSs will allow to access compounds that are not known from traditional synthetic chemistry or biology so far. As a case example we demonstrated that PKS_SgcE_ can be used to produce the natural polyene PDH (**7**), but that the enzyme is in principle also able to synthesize so far unknown multi‐branched polyenes (**8**, **9**) if our modular platform was expanded to provide methyl‐ and/or ethylmalonyl‐CoA precursors (instead of malonyl‐CoA) from CO_2_. Finally, protoyping and optimizing complex reaction networks in vitro might provide important information for the successful implementation of these pathways in vivo to create novel production strains for the synthesis of complex multi‐carbon compounds from CO_2_ in the future.^[17]^

## Conflict of interest

The authors declare no conflict of interest.

## Supporting information

As a service to our authors and readers, this journal provides supporting information supplied by the authors. Such materials are peer reviewed and may be re‐organized for online delivery, but are not copy‐edited or typeset. Technical support issues arising from supporting information (other than missing files) should be addressed to the authors.

Supplementary
