# Supplementary material for: A Modular In Vitro Platform for the Production of Terpenes and Polyketides from CO2
Source: Angew Chem Int Ed Engl. 2021 Jun 3;60(30):16420–5. doi: 10.1002/anie.202102333 (PMC8362062; doi:10.1002/anie.202102333)
Supplement: Supplementary file 1 — Supplementary [file ANIE-60-16420-s001.pdf]

Angewandte Chemie
Eine Zeitschrift der Gesellschaft Deutscher Chemiker

Supporting Information

A Modular In Vitro Platform for the Production of Terpenes and Polyketides from CO_2

*Srividhya Sundaram, Christoph Diehl, Niña Socorro Cortina, Jan Bamberger, Nicole Paczia, and Tobias J. Erb**

anie_202102333_sm_miscellaneous_information.pdf

Table of Contents

General materials and equipment 3

Experimental Procedures 3
- Chemical synthesis of CoA esters 3
- Analysis of CoA esters 3
- Table S1 3
- Table S2 3
- Plasmids, cloning and mutagenesis 3
- Protein production and purification 4
- *In vitro* reconstitution of BHAC 4
- *In vitro* reconstitution of terpenoid pathway 4
- *In vitro* reconstitution of PDH production 5
- Coupling of CETCH and BHAC 5
- Coupling of CETCH, BHAC and terpenoid pathway 5
- Analysis of CO_2 incorporation using labeled sodium bicarbonate and sodium formate 5
- UPLC-MS analysis of malate 6
- Table S3 6
- Table S4 6
- Coupling of CETCH, BHAC and PDH production 6
- UPLC-MS analysis of terpene and polyketide intermediates 6

Supplementary text 7

Optimization of CETCH, summarized from earlier publications 7

Optimization of BHAC, this work 7

Supporting figures and tables 8
- Figure S1 8
- Figure S2 8
- Figure S3 9
- Figure S4 9
- Figure S5 10
- Figure S6 10
- Figure S7 11
- Figure S8 11
- Figure S9 12
- Figure S10 12
- Table S5 13
- Table S6 14
- Table S7 15

References 17

General materials and equipment

Limonene, α-pinene and β-farnesene were obtained from Sigma Aldrich (Munich, Germany). Sabinene was obtained from Santa Cruz Biotechnology Inc. (Dallas, USA) and α-bisabolene was obtained from Alfa Aesar (Haverhill, USA). Chemicals and materials for cloning and protein expression were purchased from New England Biolabs GmbH (Frankfurt am Main, Germany) and Macharey-Nagel GmbH (Düren, Germany). Synthesis of optimized genes was done by Baseclear AG (Leiden, Netherlands). High resolution MS measurements were performed using an IDX Orbitrap High Performance Benchtop HRMS with an electrospray ion source and an Integrion HPLC system (Thermo Scientific). Mass spectroscopic data are reported as mass per charge ratio (*m/z*). Site-directed mutagenesis was performed using Quikchange II XL kit (Agilent). Identity of all recombinant proteins was confirmed using SDS-PAGE.

Experimental Procedures

Chemical synthesis of CoA esters

The synthesis of CoA esters and their analysis by LCMS was performed based on a previously published study by Peter *et al.*,[1]

Analysis of CoA esters

Malyl-CoA and acetyl-CoA were measured on a triple quadrupole mass spectrometer (Agilent Technologies 6495 Triple Quad LS/MS) equipped with an UHPLC (Agilent Technologies 1290 Infinity II) using a 50 x 2.1 mm C18 column (Kinetex 1.7 µm EVO C18 100 Å) at 25 °C. The injection volume was 2 µl of the diluted samples (1:10 in water). The flow was set to 0.250 ml/min and the separation was performed using 50 mM ammonium formate pH 8.1 (buffer A) and acetonitrile (B). We quantified the CoAs using external standard curves prepared in 1:10 diluted (water) sample matrix. The parameters for the multiple reaction monitoring (MRMs) are displayed in table S1 and the gradient in table S2. Data analysis was done using the Agilent Mass Hunter Workstation Software.

Table S1 MRM transitions for malyl- and acetyl-CoA

Compound	Precursor Ion	Product Ion	Dwell	Fragmentor	Collision Energy	Cell Accelerator Volt.	Polarity
Malyl-CoA (Quantifier)	884.1	377.1	30	380	37	5	Positive
Malyl-CoA (Qualifier)	884.1	428	30	380	29	5	Positive
Acetyl-CoA (Quantifier)	810.1	302.2	30	380	35	5	Positive
Acetyl-CoA (Qualifier)	810.1	428	30	380	35	5	Positive

Table S2 Gradient used for the separation of CoAs

Time [min]	A [%]	B [%]
0	100	0
2	100	0
5	94	6
8	77	23
10	20	80
11	20	80
12	100	0
12.5	100	0

Plasmids, cloning and mutagenesis

The plasmids generated by Schwander *et al.*,[2] and Borzyskowski *et al.*,[3] were used to produce the enzymes to reconstitute CETCH and BHAC. The plasmid for the malate dehydrogenase is derived from Kitagawa *et al.*,[4] The plasmid for the production of MtkAB is a gift from Thomas Schwander. The optimized genes corresponding to the terpene and PKS pathways were either obtained from a previous study[3] or synthesized as from Baseclear (Leiden, Netherlands). The list of all the plasmids with the details of vector and purification tags is listed in table S5. Primers and protocol for the point mutation of the PKS_{SgcE} KS domain were designed based on the Quikchange II XL mutagenesis kit manual (Agilent). The plasmid that expresses PKS_{SgcE} under the control of T7 promoter was used as template to make the mutation. The primers used for generating the point mutation are PKS_{SgcE}_KS_{Cys}_fw: CTACACGGTTGATGGCGCGGCTTCCTCTAGCTTGCTGAG and PKS_{SgcE}_KS_{Cys}_rv: CTCAGCAAGCTAGAGGAAGCCGCGCCATCAACCGTGTAG. After PCR amplification and inactivation of any template DNA by DpnI, 1/10th the sample volume of 3 M sodium acetate (pH 5.2) was added to precipitate the amplified product. The precipitate was washed with 2.5 volumes of 100% ice-cold ethanol. After a brief centrifugation, the DNA pellet was further washed with 70% ethanol. The

enriched plasmid was then directly used for transformation of *E. coli* XL-10 gold ultra-competent cells by electroporation. After confirming the mutant by DNA sequencing (Microsynth), the plasmid was introduced into *E. coli* BL21 (DE3) (New England Biolabs).

Protein production and purification

The plasmids to reconstitute the terpene and PKS pathway were expressed in *E. coli* BL21 (DE3). E. coli transformants were cultivated in LB medium at 37 °C. After A_{600nm} reached ~ 0.4 - 0.5, the cells were induced with 0.1 mM IPTG at 18 °C for 16 - 20 h. The cell pellet was dissolved (10 ml buffer/g pellet) in 150 mM Tris buffer pH 7.5 containing 0.2 M NaCl. After disrupting the cells by sonication, the cells were centrifuged at 20,000 g at 4 °C for 30 min. The lysed supernatant was then loaded onto a Ni-NTA column (Macherey Nagel) connected to a FPLC machine. Proteins were eluted using the same buffer with 0.25 or 0.5 M imidazole. For Idi, PKS_{SgcE} and TE_{SgcE}, the buffers also contained 1 mM DTT to avoid protein precipitation. The fraction containing the target protein from Ni-NTA column was diluted twice with 100 mM Tris (pH 7.5) and purified further by an ion-exchange column (5 mL HiTrap Q HP, GE Healthcare). Proteins were eluted over 20 column volumes of 100 mM Tris (pH 7.5) and 1 M NaCl, and the target proteins were concentrated using Amicon columns (MWCO 10, 30 and 100 kDa – Millipore). All the purified proteins were stored in 50 mM Tris buffer pH 7.5 containing 20 mM NaCl and 10% glycerol at -80 °C until further analysis. Except Hmgr, Idi and PKS_{SgcE}, the proteins were stable and active up to a period of 6 months under this storage condition.

Proteins to reconstitute the CO_2 to acetyl-CoA conversion (CETCH, BHAC and additional enzymes) were produced in *E.coli* BL21 (DE3) or Rosetta (DE3) pLysS (methylsuccinyl-CoA and propionyl-CoA oxidases (Mco, Pco)). For expression of 4-hydroxybutyryl-CoA synthase (Hbs) we co-expressed the 60 kDa chaperoin (groESL) for the correct folding of the protein. After transformation in the expression strains, the cultures were grown overnight on LB-agar plates containing the selection antibiotics. 2 l of salt buffered TB medium was directly inoculated with colonies from the selection plates and grown on 37 °C and 90 rpm till A_{600nm} 0.5-1.0. In general, the cultures were cooled down to 21 °C and induced with 0.25 mM IPTG. For 4-hydroxybutyryl-CoA hydratase (Hbd) 100 µM of Fe(II)SO4, 100 µM Fe(III)citrate and 20 mM fumarate were added along with IPTG. The Hbd-expressing culture was grown until A_{600nm} 4.0 and cooled down in a closed sterile Schott bottle to express the protein under microaerobic conditions. Except Pco, the expression of the proteins was done overnight. Pco was expressed at 25 °C for 4 h. The cells were harvested by centrifugation (15 min, 4 °C, 6000 g). Afterwards the cells were resuspended (2 ml buffer/g pellet) lysis buffer (500 mM NaCl, 50 mM HEPES, 10% glycerol, pH 7.8 at RT). 5 mM $MgCl_2$, 10 µg/ml DNAse and one tablet of SigmaFAST Protease Inhibitor Cocktail (Sigma-Aldrich) were added. The cells were lysed by micro fluidizer (twice at 16.000 psi). Afterwards the cell debris was spun down at 50,000 g for 1 h at 4 °C. The supernatant was filtered through a 0.45 µm membrane. Except for the glyoxylate reductase, the lysate was mixed with 3 ml Protino Ni-NTA agarose beads (Macherey-Nagel) and incubated on ice for 30-45 min (70 rpm). Afterwards the beads were collected in a gravity column and washed with three column volumes (cv) of lysis buffer. For the removal of unspecific bound proteins, the beads were washed with three cv of lysis buffer containing an additional 50 mM of imidazole and three cv with 75 mM imidazole. The elution was done with two cv of lysis buffer containing 500 mM imidazole. Since the glyoxylate reductase has a streptavidin (Strep) tag, the lysate was loaded on a Cytiva StrepTrap™ HP prepacked column attached to an Äkta start FPLC adjusted to a flow rate of 1 ml/min. The desalting/storage buffer was used for lysis and purification. The elution was done using the desalting/storage buffer with 5 mM d-Desthiobiotin. The collected recombinant proteins were concentrated using Amicon Ultra 15 mL Centrifugal Filters (Merck) accordingly to the protocol provided by the supplier. For desalting the protein solution was loaded on a HiLoad 16/600 Superdex 200 pg column (GE Healthcare). The desalting/storage buffer contained 200 mM NaCl, 50 mM HEPES and 10% glycerol and was adjusted to pH 7.8 at room temperature (22 ˚C). For Hbs and Hbd a concentration of 500 mM NaCl was used. The collected fractions were pooled and concentrated again. FAD was added to Pco and Mco depending on the concentration of protein. Enzymes requiring metal ions and cofactors were stored in 5 mM $MgCl_2$ and 2 mM Coenzyme B12 respectively. For the final storage, glycerol was added to a final concentration of 20 %. The proteins were flash frozen in liquid nitrogen and stored at -80 °C until further analysis.

***In vitro* reconstitution of BHAC**

We tested four different setups for the reconstruction of the whole BHAC (**Figure S1A**). The general assay mix contained 100 mM HEPES-KOH pH 7.5, 5 mM $MgCl_2$, 20 mM sodium formate, 5 mM NADH, 5 mM NADPH, 0.1 mM pyridoxalphosphate, 14.4 µM (0.67 mg/ml) formate dehydrogenase, 0.33 µM (0.0115 mg/ml) malate dehydrogenase, 2.26 µM (0.099 mg/ml) BhcB, 1.37 µM (0.049 mg/ml) BhcC, 14.84 µM (0.508 mg/ml) BhcD and 0.5 mM glyoxylate as substrate. The four setups contained additionally: 1) 0.5 mM glycine and 0.79 µM (0.043 mg/ml) BhcA, 2) 5.0 mM glycine and 0.79 µM (0.043 mg/ml) BhcA, 3) 0.5 mM glycine and 19.83 µM (0.890 mg/ml) BhcA and 4) 5.0 mM glycine and 19.83 µM (0.890 mg/ml) BhcA. The reactions were carried out at 30 °C in duplicates and in 50 µl reaction volume. 12 µl samples were withdrawn at 30, 60 and 90 min and quenched with 1.5 µl of 50 % formic acid and 1.5 µl of 500 mM polyphosphate for protein precipitation. The quenched samples were kept on ice until the end of the experiment and spun down at 20,000 g for 20 min at 4 °C. The supernatant was transferred into fresh tubes and stored at -20 °C until measurement.

***In vitro* reconstitution of the terpene biosynthesis modules**

The reactions for the *in vitro* production of **1-5** were performed in a sealed glass vial. Briefly, in a 100 µL reaction, 1 mM NADPH, 20 mM formate, 3 mM ATP, 3 mM PEP, 2 mM NADH, 1 mM DTT, 5 mM $MgCl_2$, 10 mM KCl were added in 50 mM Tris buffer pH 8.0. The list of enzymes and their amounts are listed in table S5. The reaction was initiated by adding 0.5 mM or 1 mM acetyl-CoA. To trap

the volatile monoterpenes (**1-3**), the assay mix was overlayed with 30 µl of isopropylmyristate. The samples were incubated at 30 °C with shaking at 400 rpm up to 24 h. At specified intervals, the organic layer is withdrawn and diluted with hexane. The volume of isopropylmyristate withdrawn was simultaneously added to the reaction mix during the course of the assay. For the samples assaying the production of **4** and **5**, at these intervals, the workup of the samples was done by extracting twice the volume with ethyl acetate. The mix was then spun at 20,000 g for 15 min at 4 °C. The aqueous phase was mixed with equal volume of methanol and centrifuged to precipitate the proteins. Both the organic and aqueous phase were saved at -80 °C until further analysis. All the reactions were set up in triplicates.

***In vitro* reconstitution of the PDH production**

The *in vitro* assay for the reconstitution of PKS pathway to produce pentadecaheptaene (PDH) was performed as described previously[5] with minor modifications. In 100 mM phosphate buffer pH 8.0, 0.2 mM or 1.2 mM acetyl-CoA, 1.2 mM malonyl CoA, 1.2 mM NADPH, 1 mM DTT, 8 mM $MgCl_2$, 40 mM $KHCO_3$, 1 mM ATP, 0 to 10 µM PKS_{SgcE}, 0 to 50 µM TE_{sgcE} and 2 µM Pcc* were added to a total of 200 µl. The list of enzymes and their amounts are listed in table S5. The assay was performed at 30 °C with shaking at 400 rpm up to 24 h. At specified intervals, the sample was withdrawn and the polyketides are extracted twice the volume with ethyl acetate. After evaporating the organic layer with an upstream flow of nitrogen, the residue was dissolved in 100 µl ethyl acetate. For UV-Vis analysis, the extract was measured at 395 nm to detect the formation of pentadecaheptaene **7**. After adding equal volume of methanol to the aqueous layer to precipitate proteins, the mix was spun down at 20,000 g at 4 °C for 10 min. The samples were stored at -80 °C until further analysis. All the reactions were set up in triplicates.

Coupling of CETCH and BHAC

For the coupling of the CETCH with the BHAC we used the same enzyme concentrations of the CETCH core cycle as in[2] For the BHAC enzymes and the Mdh we used the amounts as described in ***In vitro* reconstitution of BHAC** I) above, except for the BhcD where the amount was increased by a factor of five. MtkAB was added at a concentration of 13.54 µM (1 mg/ml) and the glyoxylate reductase at a concentration of 0.62 µM (0.020 mg/ml). Other components were added in the following concentrations: 5 mM $MgCl_2$, 20 mM polyphosphate, 50 mM sodium bicarbonate, 20 mM sodium formate, 1 mM coenzyme A, 0.1 mM coenzyme B_{12}, 5 mM ATP, 5 mM NADPH, 5 mM NADH, 1 mM glycine, 0.1 mM pyridoxalphosphate and 100 µM propionyl-CoA as substrate. For the positive control (**Figure S1B (a)**), the enzymes of the CETCH core cycle were used together with the glyoxylate reductase to produce glycolate. To produce acetyl-CoA the enzymes of the CETCH core cycle were combined with BHAC enzymes, and Mdh plus MtkAB (**Figure S1B (c)**). The assays were done in triplicates at 50 µl each. 13.5 µl samples were taken at 60, 120 and 180 min and quenched in 1.5 µl 50% formic acid to stop the reaction. For the split assay, the CETCH core enzymes were used to produce glyoxylate for 60 min. The assay was done in a single assay, split into two batches after 60 min where either the glyoxylate reductase (**Figure S1B (b)**) or the BHAC enzymes, Mdh and MtkAB were added (**Figure S1B (d)**) and then further divided in triplicates to 50 µl. Samples were taken as described before at 120 and 180 min. The quenched samples were kept on ice until the end of the experiment and spun down at 20,000 g for 20 min at 4 °C. The supernatant was transferred into fresh tubes and stored at -20 °C until measurement.

Coupling of CETCH, BHAC and terpene biosynthetic modules

The coupling assay was performed in 2 steps by preparing the CETCH-BHAC and the terpene assay mix separately and then mixing equal volume (50 µl) of both in one-pot. In 100 mM Hepes buffer pH 7.5, the CETCH-BHAC assay mix contained 5 mM ATP, 5 mM NADPH, 5 mM NADH, 5 mM $MgCl_2$, 20 mM polyphosphate, 50 mM bicarbonate, 20 mM formate, 1 mM CoA, 0.1 mM vitamin B_{12}, 1 mM glycine, 0.1 mM PLP, and the enzymes with amounts specified in table S5. In 50 mM Tris pH 8.0, the terpenoid assay mix contained 20 mM formate, 3 mM PEP, 10 mM KCl together with the enzymes listed in table S5 including 80 µg limonene synthase, 60 µg sabinene synthase, 80 µg α-pinene synthase, 40 µg α-bisabolene synthase, 40 µg β-farnesene synthase. After mixing both the mixes to 100 µl, the reaction was started with 0.1 mM propionyl-CoA and were incubated at 30 °C with shaking at 400 rpm up to 24 h. As positive controls, the CETCH-BHAC and the terpene assays were performed in parallel by adding 0.5 mM acetyl CoA to the latter. At regular intervals, samples were withdrawn from both the positive controls and the tests. Work-up of the samples to detect **1-5** was performed as described in ***In vitro* reconstitution of the terpene biosynthesis modules**. All the reactions were set up in triplicates.

Analysis of CO_2 incorporation using ^{13}C-labeled sodium bicarbonate and sodium formate

To verify the incorporation of CO_2 by the CETCH cycle as described in Schwander *et al.*,[2] we performed the CETCH-BHAC coupling (**Figure 1C**) with 50 mM ^{13}C-labeled sodium bicarbonate (and carbonic anhydrase) and 20 mM ^{13}C-labeled sodium formate. ^{13}C-labeled sodium formate was used to derive $^{13}CO_2$ released by the formate dehydrogenase for NADPH regeneration. All the other components that were present are described in **Coupling of CETCH and BHAC** and the sampling procedure remained the same**.** Malate-CoA ligase was ommited to produce malate as the final readout. The reaction was started with either 100 µM propionyl-CoA (positive control) or ddH_2O (negative control). For the evaluation by LC-MS, we used a targeted method to quantify the decarboxylated fragment of malate.

UPLC-MS analysis of malate

The different fragments of ^{13}C-labeled malate were measured on a triple quadrupole mass spectrometer (Agilent Technologies 6495 Triple Quad LS/MS) equipped with an UHPLC (Agilent Technologies 1290 Infinity II) using a 150 x 2.1 mm C18 column (Kinetex 1.7 µm EVO C18 100 Å) at 25 °C. The injection volume was 1 µl of the diluted samples (1:25 in water). The flow was set to 0.100 ml/min and the separation was performed using dH_2O with 0.1% formic acid (buffer A) and methanol with 0.1% formic acid (B). Since malate is a dicarboxylic acid and it was unclear which carboxylic group leaves the molecule, we measured all the possible transitions The parameters for the multiple reaction monitoring (MRMs) are displayed in table S3 and the gradient in table S4. Data analysis was done using the Agilent Mass Hunter Workstation Software.

Table S3 MRM transitions for decarboxylated fragment of malate

Compound	Precursor Ion	Product Ion	Dwell	Fragmentor	Collision Energy	Cell Accelerator Volt.	Polarity
Malate +0 (Quant.)	133	89	35	380	11	5	Negative
Malate +0 (Qual.)	133	133	35	380	0	5	Negative
Malate +1 (Quant.)	134	89	35	380	11	5	Negative
	134	90	35	380	11	5	Negative
Malate +1 (Qual.)	134	134	35	380	0	5	Negative
Malate +2 (Quant.)	135	90	35	380	11	5	Negative
		91	35	380	11	5	Negative
Malate +2 (Qual.)	135	135	35	380	0	5	Negative
Malate +3 (Quant.)	136	91	35	380	11	5	Negative
	136	92	35	380	11	5	Negative
Malate +3 (Qual.)	136	136	35	380	0	5	Negative
Malate +4 (Quant.)	137	92	35	380	11	5	Negative
Malate +4 (Qual.)	137	137	35	380	0	5	Negative

Table S4 Gradient used for the measurement of the decarboxylated fragments of malate

Time [min]	A [%]	B [%]
0	100	0
4	100	0
6	0	100
7	0	100
7.1	100	0
12	100	0

Coupling of CETCH, BHAC and PDH production

In 100 mM phosphate buffer pH 8.0, 40 mM $KHCO_3$, 2.5 µM PKS_{SgcE}, 40 µM TE_{SgcE} and 2 µM Pcc* were added to a total of 50 µl. The list of enzymes and their amounts are listed in table S5. The reaction was initiated by adding equal volume of CETCH-BHAC mix (test) or 1.2 mM acetyl CoA (positive control). The assay was performed at 30 °C with shaking at 400 rpm up to 24 h. At specified intervals, the sample was withdrawn and the polyketides were extracted twice the volume with ethyl acetate. After evaporating the organic layer with an upstream flow of nitrogen, the residue was dissolved in 100 µl ethyl acetate. After adding equal volume of methanol to the bottom aqueous layer to precipitate proteins, the mix was spun down at 20,000 g at 4 °C for 10 min. All the reactions were set up in triplicates.

UPLC-MS analysis of terpene and polyketide intermediates

Analysis of the all the terpenes was done in GCMS (Agilent 5973N/6890N single quadrapole) by measuring 1 µL of the samples. An OPTIMA 5 column (30 m long, 0.32 mm inner diameter, 0.25 µm thick) was used for the separation with an initial temperature of 60 °C (2 min hold) followed by a gradient from 20 °C (1 min) to 150 °C then from 40 °C (1 min) to 320 °C. A constant flow rate of 1 ml/min was used. The injector had a temperature of 210 °C and was set for a 1:25 split. The MS had a mass range from 34 to 550 Da covered. The aqueous phase from the independent and coupled terpene experiments was directly analysed for the isoprenoid intermediates using UPLC-high resolution mass spectrometer (Orbitrap IDX^{TM}) set to negative ionisation mode. SeQuant ZIC-pHILIC (150 x 4.6 mm) was used for separating the isoprenoid intermediates. UPLC conditions: isocratic elution (10 mM ammonium carbonate and 118 mM ammonium hydroxide in acetonitrile:water (60.1:39.8)) for 10 min at a flow rate of 0.45 ml/min; injection volume: 3 µL; mass range: 65 – 1100 *m/z*). For the analysis of polyketides, both the organic and aqueous phased were analysed directly using UPLC-high resolution mass spectrometer (Orbitrap IDX^{TM}) set to positive ionisation mode. Kinetic EVO C18 column (50 x 2.1 mm) was used for the separation of the polyketide intermediates. UPLC conditions: 95 % of 0.1 % formic acid in water (Solvent A) for 2 min; 5 – 95 %

0.1 % formic acid in acetonitrile (Solvent B) for 2 – 11 min; 95 % B at 12 min; 95 % until 14 min. flow rate: 0.25 ml/min; injection volume: 5 µL; mass range: 100 – 1100 *m/z*).

Supplementary text

Optimization of CETCH, summarized from earlier publications

CETCH had been optimized earlier in several rounds (CETCH 1.0 to CETCH 5.4).[2] All versions of CETCH were tested in buffer containing 50 mM sodium bicarbonate which was further equilibrated with carbonic anhydrase to provide CO_2 in a dissolved form to the assay. In CETCH 2.0, methylsuccinyl-CoA dehydrogenase was engineered into a methylsuccinyl-CoA oxidase (Mco) to catalyze the oxidation of methylsuccinyl-CoA with molecular oxygen, which allowed CETCH to turn multiple times as shown by ^{13}C-labeling experiments. In CETCH 3.0, a read-out module was introduced to convert glyoxylate to malate that allowed a better quantification of CETCH assays. Also, an engineered formate dehydrogenase was used to regenerate the cofactor NADPH and (simultaneously) CO_2 in the assay. In CETCH 4.0, to protect the cofactors and intermediates and to prevent the oxidative damage from H_2O_2 (produced by Mco), catalase (KatE) was added which resulted in increased efficiency in CO_2 fixation. In CETCH 5.0, to maintain a stable ATP pool and to regenerate ATP for Hbs, a polyphosphate transferase (Ppk) was included that increased the efficiency of the cycle to fix 4.3 CO_2-equivalents per acceptor molecule in 90 min. Finally, after further improvements to the cycle (optimizing Ccr), the efficiency of CETCH 5.4 reached a maximum of 5.4 fixed CO_2-equivalents per acceptor in 90 min. The CETCH cycle reached a plateau after 90 min and malate production could not be increased beyond 540 µM, indicating that malate inhibits CETCH cycle enzymes.

Optimization of BHAC, this work

To establish and optimize the BHAC, we reconstituted the BHAC *in vitro* using N-terminal His-tagged proteins, produced in *E. coli*. To test the functioning of the BHAC cycle, we started the reaction with 500 µM glyoxylate and monitored the formation of malate from oxaloacetate, using malate dehydrogenase (Mdh) over time. Note that β-hydroxyaspartate aldolase (BhcC), the first enzyme reaction of the BHAC that catalyzes the aldol condensation of glyoxylate with glycine, has an apparent Km of 4.3 ± 0.3 mM for glycine.[3] Thus, while providing high glycine concentrations might facilitate the first reaction, it might also lead to a faster depletion of glyoxylate, which is required in the last step as acceptor for aspartate-glyoxylate aminotransferase (BhcA), eventually creating a bottleneck. To optimize BHAC productivity, we initially tested two different glycine (0.5 mM and 5 mM), as well as two different BhcA (0.79 µM and 19.83 µM) concentrations. However, over the course of 90 min, total malate yields were comparable between the different conditions tested (~70%), indicating that the BhcC was operating robustly across a wide range of co-substrate and BhcA concentrations *in vitro* (**Figure S1A**).

Supporting Figures and Tables

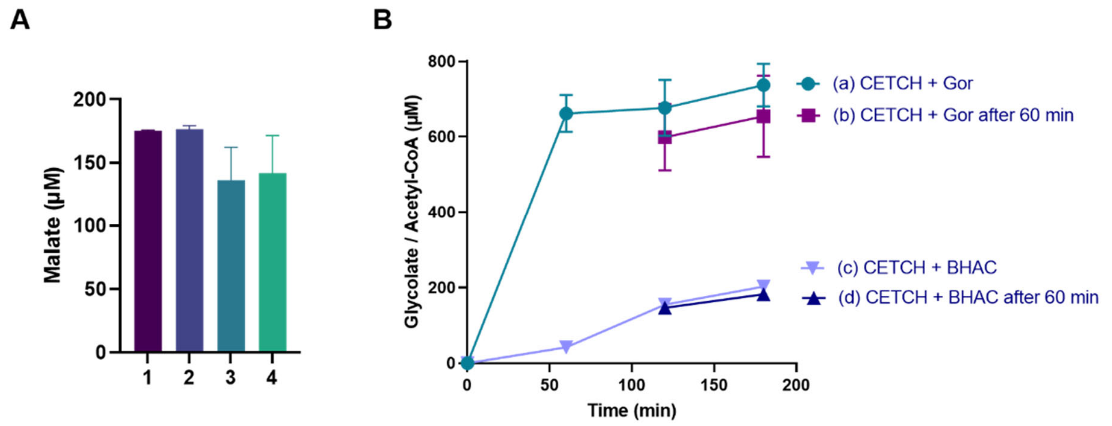

Figure S1 BHAC reconstitution and coupling to CETCH cycle for acetyl-CoA production. **A)** Malate production by the BHAC after 90 min. The general setup is described in: ***In vitro* reconstitution of BHAC.** All the reactions were started with 500 µM glyoxylate. With this setup, we tested different BhcA and glycine concentrations: 1) 0.5 mM glycine and 0.79 µM (0.043 mg/ml) BhcA, 2) 5.0 mM glycine and 0.79 µM (0.043 mg/ml) BhcA, 3) 0.5 mM glycine and 19.83 µM (0.890 mg/ml) BhcA and 4) 5.0 mM glycine and 19.83 µM (0.890 mg/ml) BhcA. The data represent n=2 ± standard deviation. **B)** Acetyl-CoA vs. glycolate production by the CETCH (+BHAC). The general setup is described in: **Coupling of CETCH and BHAC.** All the reactions were started with 100 µM propionyl-CoA. In this setup we tested whether the low acetyl-CoA yield is due to interference of the BHAC enzymes with the core cycle or due to intermediate drainage. (a) CETCH core cycle with Glyoxylate reductase (Gor). (b) Glyxoylate reductase added after 60 min. (c) BHAC enzymes, Mdh and MtkAB added after 60 min. (d) CETCH with BHAC enzymes, Mdh and MtkAB. The data represent n=3 ± standard deviation.

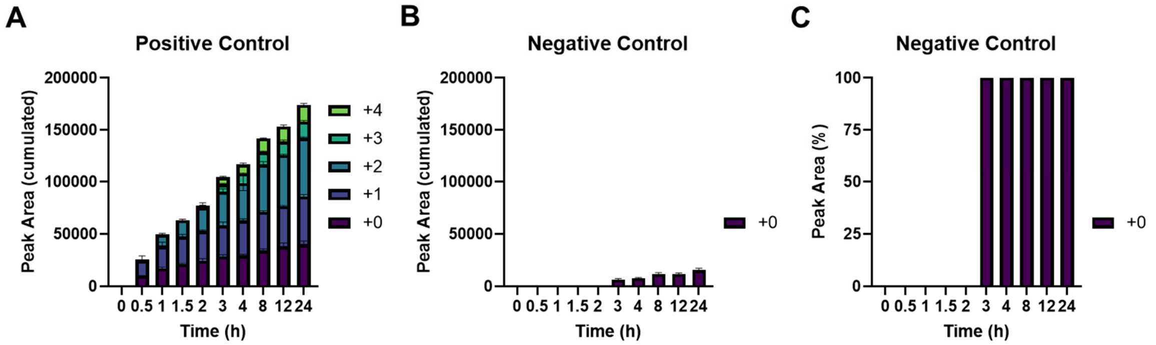

Figure S2 Fractional labeling of malate by incorporation of $^{13}CO_2$. +0, +1, +2, +3, +4 indicates the number of ^{13}C are incorporated into malate. To verify the incorporation of CO_2 by the CETCH cycle we repeated the CETCH-BHAC coupling (**Figure 1C**) with 50 mM ^{13}C-labeled sodium bicarbonate (and carbonic anhydrase) and 20 mM ^{13}C-labeled sodium formate. ^{13}C-labeled sodium formate was used to derive $^{13}CO_2$ released by the formate dehydrogenase for NADPH regeneration. In the first three turns of the CETCH cycle only single labeled glyoxylate is produced while the second $^{13}CO_2$ derived carbon is incorporated into CETCH cycle intermediates.[2] For the formation of oxaloacetate and therefore malate by the BHAC, initially added glycine is used. Since the last reaction in the BHAC for the production of oxaloacetate requires another molecule of glyoxylate generated from fixed CO_2, a single labeled molecule of malate is stoichiometrically completely build from fixed CO_2. **A**) Total level of malate dissected into the labeled fractions. For fractional labeling of the positive control see **Figure 1D**. **B**) Fractional labeling in percentage of the negative control containing buffer. **C**) Total malate in the negative control. The data represent n=3 ± standard deviation.

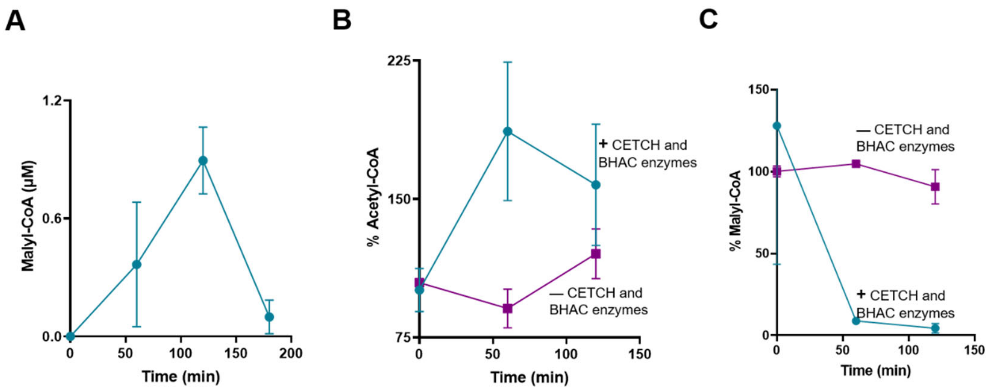

Figure S3 Profile of CoA esters in the CETCH-BHAC coupled assay. **A**) Residual concentration of malyl-CoA in the assay shown in Figure S1B (c). Stability of **B**) acetyl-CoA and **C**) malyl-CoA under assay conditions described in **Coupling of CETCH and BHAC**. All enzymes and cofactors except Mcl (to avoid the cleavage of malyl-CoA) was added to the positive control (**+**). Only the cofactors are added to the negative control (—). 100% corresponds to 300 µM of acetyl- and malyl-CoA. While acetyl-CoA was stable, malyl-CoA was depleted in less than 60 min. The data represent n=2 ± standard deviation.

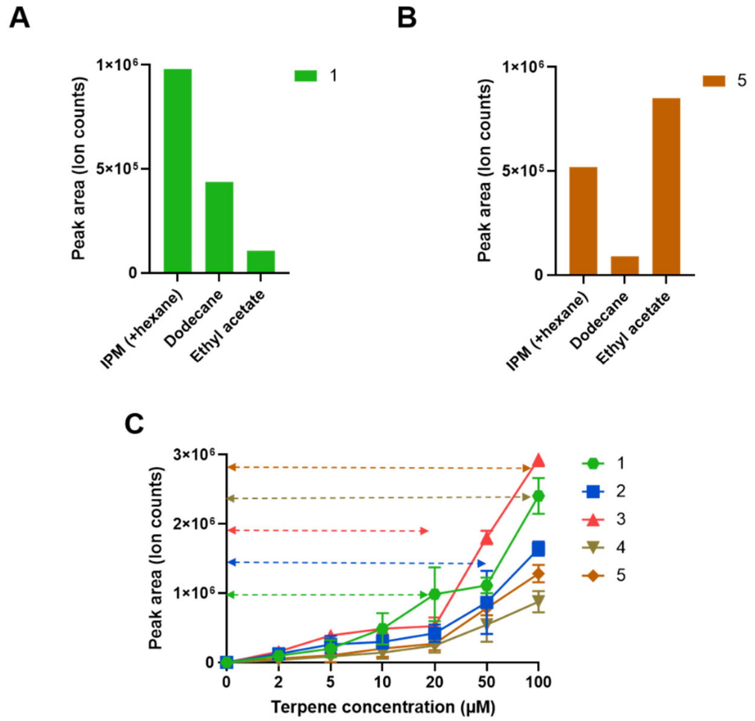

Figure S4 Optimization of terpene extraction in different solvents as measured by GCMS. Comparison of yield of **A**) 50 µM limonene (**1**) (representation) and **B**) 50 µM β-farnesene (**5**) (representation) with different organic solvents. IPM: isopropylmyristate. 30 µl IPM was added as an overlay to a 100µl standard + buffer mix. After a brief incubation, the IPM layer was carefully withdrawn and diluted with hexane before measurement using GCMS. Similarly, 10 % dodecane was also tested as an overlay to trap the terpenes. The withdrawn dodecane layer was further diluted with ethyl acetate for GCMS measurement. As a third solvent, 2x volumes of ethyl acetate (200 µl to a 100 µl standard + buffer mix) was tested. Followed by centrifugation at 20,000 g for 15 min at 4 °C, the organic phase was directly used for GCMS measurement. IPM resulted in maximum trapping of monoterpenes **1-3** which was routinely used for subsequent measurements. For sesquiterpenes **4-5**, ethyl acetate was the best solvent. **C**) Measurement of terpene standards by GCMS. The linear range used for quantification of the corresponding terpenes is shown as dashed double-arrowed lines.

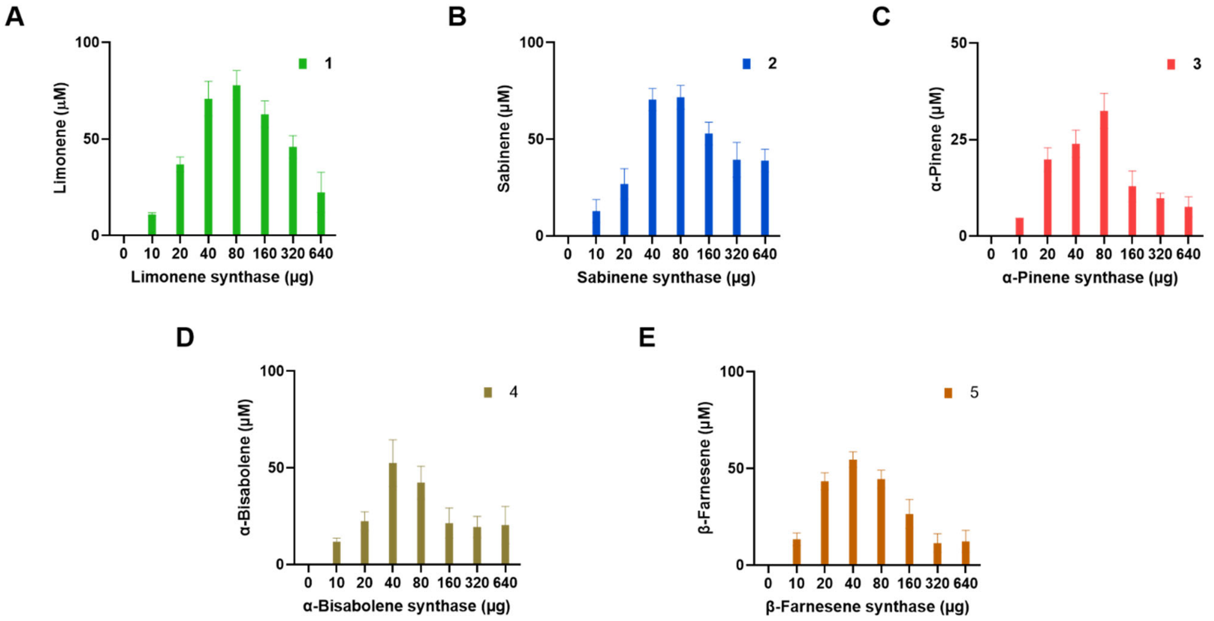

Figure S5 Comparison of different terpene synthase concentration (0 to 640 µg) for the production of terpenes and measurement by GCMS. The reaction is started with 0.5 mM acetyl CoA and run at 30 °C for 24 h. The extraction of monoterpenes **1-3** was done using isopropylmyristate overlay followed by dilution with hexane while sesquiterpenes **4-5** were extracted using 2x volumes of ethylacetate. The concentration of individual terpenes was quantified using the standard graph (**Figure S4C** linear range). The concentration at which maximum terpene production was observed has been chosen for the subsequent analysis.

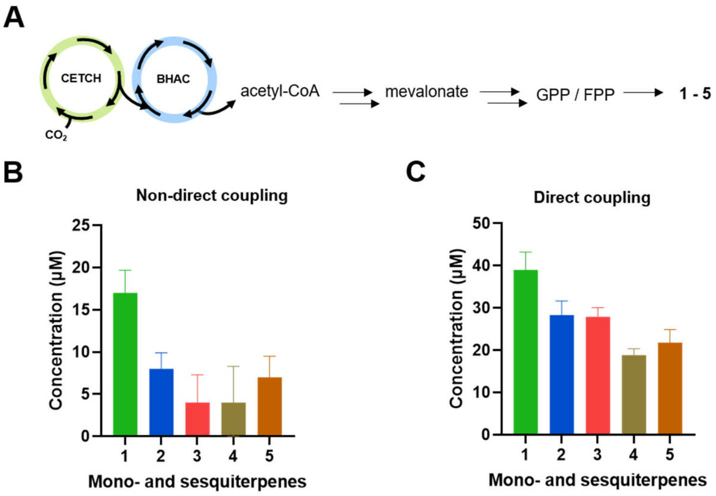

Figure S6 A) General scheme of the assay as described in methods section '**Coupling of CETCH, BHAC and terpene biosynthetic modules'**. Net production of terpenes in non-direct vs. direct coupling assay in 24 h. **B**) The CETCH-BHAC assay is first run independently for 4 h to which the terpene assay mix was subsequently added. In this non-direct coupling assay, the overall yield of monoterpenes **1-3** and sesquiterpenes **4-5** were below 20 µM. **C**) CETCH-BHAC cascade and terpene biosynthesis modules were operated in a single pot continuously. In this direct coupling approach, the net yield of terpenes improved 3- to 4-fold.

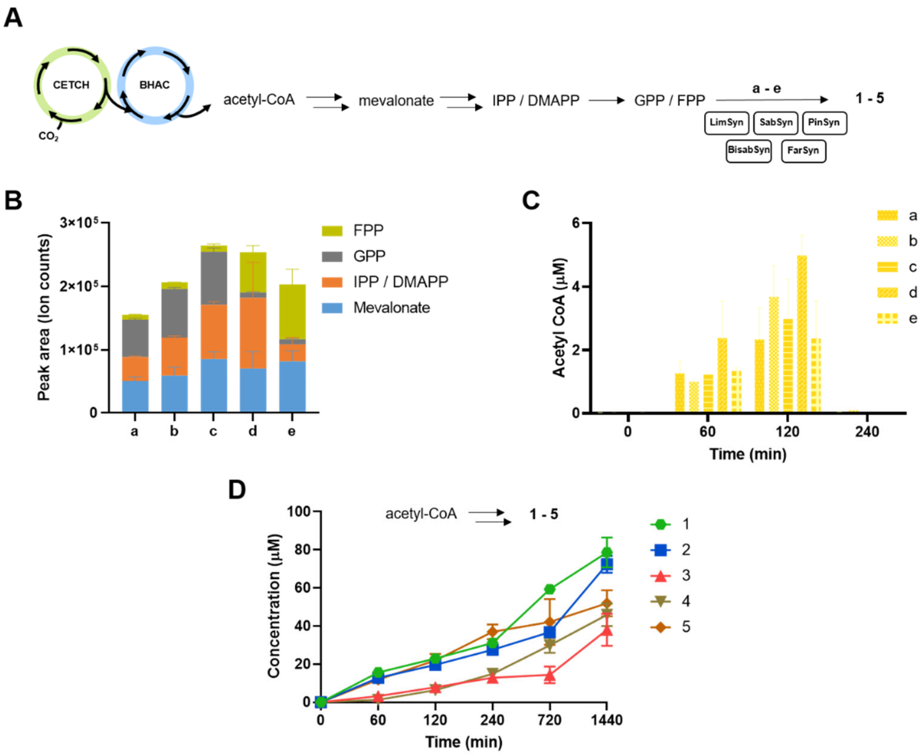

Figure S7 A) General scheme of the direct coupling assay as described in methods section '**Coupling of CETCH, BHAC and terpene biosynthetic modules'**. a, b, c, d, e refers to individual reactions with limonene synthase, sabinene synthase, α-pinene synthase, α-bisabolene synthase and β-farnesene synthase respectively. **B**) Accumulation of mevalonate pathway intermediates measured by LCMS at 24 h. Equal volume of methanol was added to the final assay mix to stop the reaction and to precipitate the proteins. After centrifugation at 20,000 g for 15 min at 4°C, the supernatant was used directly to measure by LCMS. **C**) Concentration of residual acetyl-CoA measured over 4 h. From 4 h, only negligible amounts of acetyl-CoA could be detected. **D**) Acetyl CoA to terpenes as a positive control. The reaction is started with 0.5 mM acetyl-CoA.

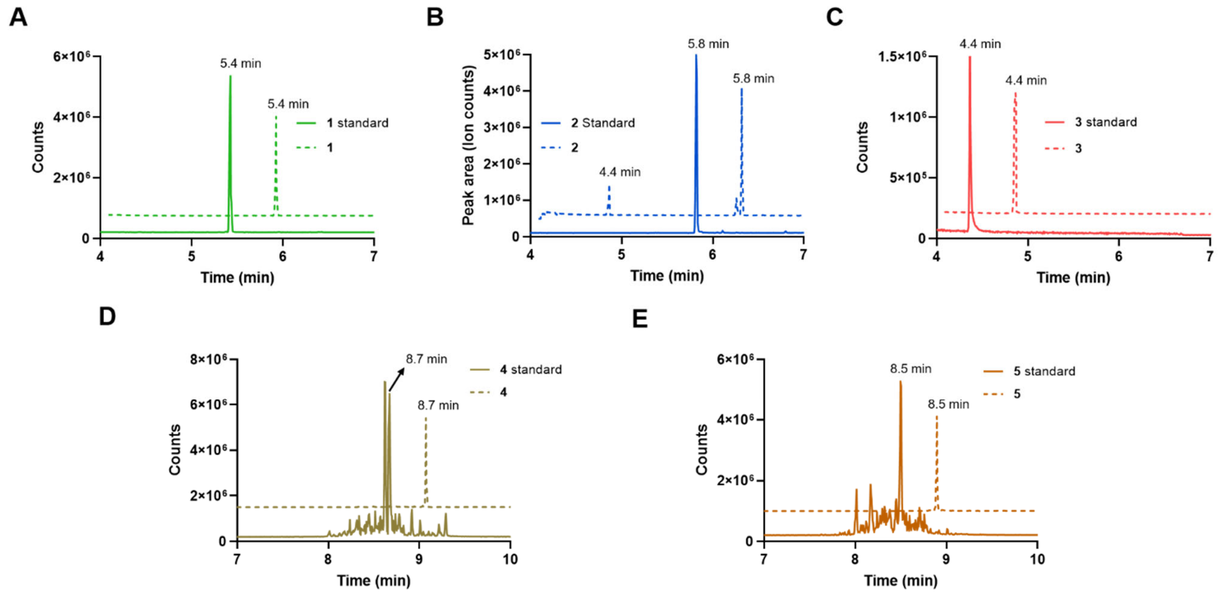

Figure S8 GCMS analysis of the production of mono- and sesquiterpenes from the CETCH-BHAC-terpene coupled assay (refer figure 2C) and comparison with authentic standards. The assay is performed as described in the methods section '**Coupling of CETCH, BHAC and terpene biosynthetic modules'**. For clarity, only the traces at 24 h time point are shown. **A**) Representative trace of limonene (**1**) from 100 μM propionyl-CoA. **B**) Representative trace of sabinene (**2**). A fraction of α-pinene (**3**) was also observed at a retention time of 4.4 min. **C**) Representative trace of α-pinene (**3**). **D**) Representative trace of α-bisabolene (**4**). The bisabolene standard come as a mixture of isomers however, exclusively α-bisabolene is observed in the GCMS trace in the assay sample. **E**) Representative trace of β-farnesene (**5**).

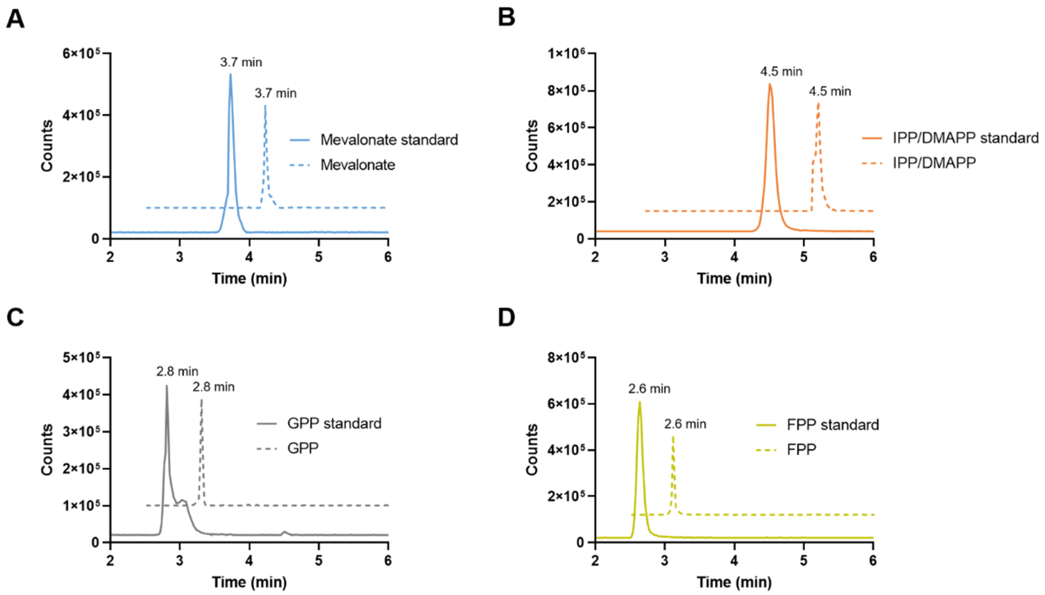

Figure S9 LCMS analysis of the terpene intermediates from the CETCH-BHAC-terpene coupled assay (refer figure 2C) and comparison with authentic standards. Representative traces of **A**) mevalonate, **B**) IPP/DMAPP, **C**) GPP and **D**) FPP. IPP and DMAPP could not be separated even after optimizing the chromatographic method. Equal volume of methanol was added to the final assay mix (and the standards, as a positive control) to stop the reaction and to precipitate the proteins. After centrifugation at 20,000 g for 15 min at 4°C, the supernatant was used directly to measure by LCMS.

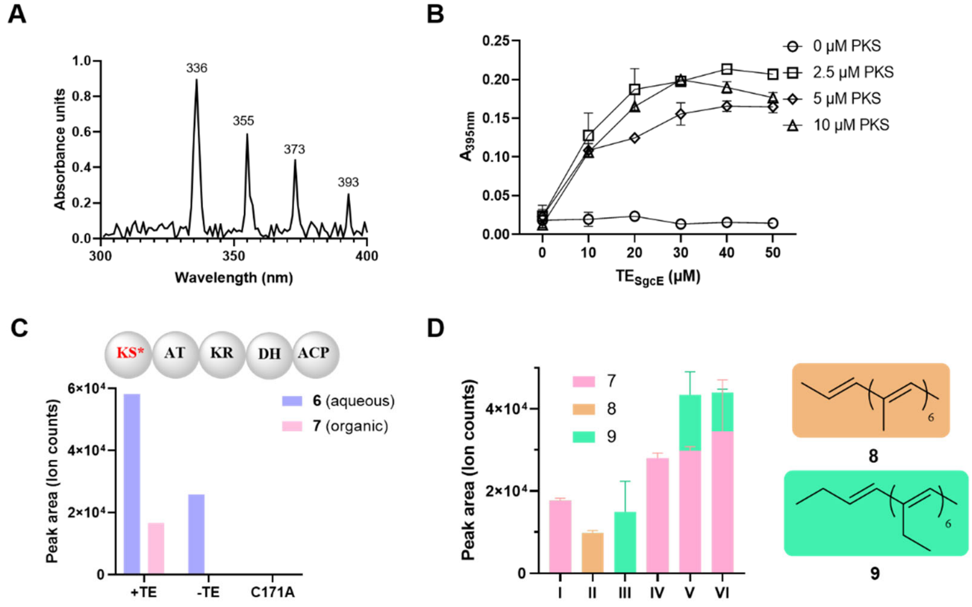

Figure S10 A) UV-Vis profile of pentadecaheptaene (**7**). **7** exhibited a spectrum typical of a polyene with multiple absorption maxima between 300 and 400 nm. **B**) Absorbance of the ethyl acetate extracts from the PKS enzymatic assay at 395 nm. The production of **7** at various PKS_{SgcE} and TE_{SgcE} concentrations is shown. 2.5 μM PKS_{SgcE} and 40 μM PKS_{TE} was used for the subsequent analysis. **C**) Analysis of the KS_{C171A} mutant. Compared to the positive control (+TE), neither the production of **6** nor **7** was observed. **D**) Formation of substituted heptaenes (**8** and **9**) from 100 μM propionyl-CoA in the CETCH-BHAC-PKS coupled assay, using different extender units. I: malonyl-CoA (positive control); II: methylmalonyl-CoA; III: ethylmalonyl-CoA; IV: malonyl- + methylmalonyl-CoA; V: malonyl- + ethylmalonyl-CoA; VI: malonyl- + methylmalonyl- + ethylmalonyl-CoA. All the assays were performed in triplicates and the mean ± S.D. are plotted.

Table S5. List of enzymes used in the study

Cycle/pathway	Abbreviation	Full name	Source	Vector	Tag	Origin Reference
CETCH	Pco	Propionyl-CoA oxidase	*A. thaliana*	pET16b	His	[2]
CETCH	Ccr	Crotnonyl-CoA carboxylase/reductase	*M. extorquens*	pET16b	His	[2]
CETCH	Epi	Epimerase	*R. sphaeroides*	pET16b	His	[6]
CETCH	Mcm	Methylmalonyl-CoA mutase	*R. sphaeroides*	pET16b	His	[7]
CETCH	Scr	Succinyl-CoA reductase	*C. kluyveri*	pCDF-Duet-1	His	[2]
CETCH	Ssr	Succinic semialdehyde reductase	*H. sapiens*	p2BP1	His	[2]
CETCH	Hbs	4-hydroxybutyryl-CoA synthetase	*N. maritimus*	pET16b	His	[8]
CETCH	Hbd	4-hydroxybutyryl-CoA dehydratase	*N. maritimus*	pRSET-B	His	[2]
CETCH	Ecm	Ethylmalonyl-CoA mutase	*R. sphaeroides*	pET16b	His	[6]
CETCH	Mco	Methylsuccinyl-CoA oxidase	*R. sphaeroides*	pET16b	His	[2]
CETCH	Mch	Mesaconyl-CoA hydratase	*R. sphaeroides*	pET16b	His	[9]
CETCH	Mcl	Malyl-CoA/citramalyl-CoA lyase	*R. sphaeroides*	pET16b	His	[10]
CETCH	KatE	Catalase	*E. coli*	pCAN24N (ASKA JW1721)	His	[4]
CETCH	Fdh	Formate dehydrogenase (D221A)	*M. vaccae*	pET21a	His	[11]
CETCH	smPPK2-I	Polyphosphate kinase ADP - ATP	*S. meliloti*	pET28a	His	[2]
CETCH	Gor	Glyoxylate/sucinnic semialdehyde reductase	*G. oxidans*	pTE1125	Strep	Gift from Martina Carrillo Camacho
BHAC	BhcA	Aspartate glycine aminotransferase	*P. denitrificans*	pET16b	His	[3]
BHAC	BhcD	Iminsuccinate reductase	*P. denitrificans*	pET16b	His	[3]
BHAC	BhcB	Beta-hydroxyaspartate dehydratase	*P. denitrificans*	pET16b	His	[3]
BHAC	BhcC	Beta-hydroxyaspartate aldolase	*P. denitrificans*	pET16b	His	[3]
BHAC	Mdh	Malate dehydrogenase	*E. coli*	pCAN24N (ASKA JW3205)	His	[4]
BHAC	MtkAB	Malate thiokinase	*M. extorquens*	pET28b	His	Gift from Thomas Schwander
Terpene	PhaA	Acetyl‐CoA acetyltransferase	*C. necator*	pET28a	His	[12]
Terpene	Hmgs	HMG-CoA synthase (A110G)	*E. faecalis*	pET28a	His	[12]
Terpene	Hmgr	HMG-CoA reductase	*E. faecalis*	pET28a	His	[12]
Terpene	Mvk	Mevalonate kinase	*M. mazei*	pET28a	His	[12]
Terpene	Pmvk	Phosphomevalonate kinase	*S. pneumoniae*	pET28a	His	[12]
Terpene	Mdc	Mevalonate-PP decarboxylase	*S. pneumoniae*	pET28a	His	[12]
Terpene	Idi	Isopentenyl-PP isomerase	*E. coli*	ASKA JW2857	His	[4]
Terpene	Gpps	Farnesyl-PP synthase (S82F)	*G. stearothermophilus*	pET28a	His	[12]
Terpene	IspA	Farnesyl-PP synthase	*E.coli*	pET28a	His	[4]
Terpene	LimSyn	(+)-Limonene synthase	*M. spicata*	pET28a	His	[12]
Terpene	SabSyn	Limonene synthase (N345A)	*M. spicata*	pET28a	His	[12]

Terpene	PinSyn	α-Pinene synthase	*P. sitchensis*	pET28a	His	[12]
Terpene	BisabSyn	α-Bisabolene synthase	*A.grandis*	pET28a	His	Synthesized gene
Terpene	FarSyn	β-Farnesene synthase	*M.piperita*	pET28a	His	Synthesized gene
Terpene	PK/LDH	Pyruvate kinase/lactate dehydrogenase	*Sigma*			
PKS	PKS_{SgcE}	C-1027 polyketide synthase	*S.globisporus*	pET28a	His	Synthesized gene
PKS	TE_{SgcE}	C-1027 thioesterase	*S.globisporus*	pET28a	His	Synthesized gene
PKS	Pcc*	Propionyl-CoA carboxylase (D407I)	*M. extorquens*	JZ105	His	Gift from Jan Zarzycki

Table S6. Kinetic data of enzymes

Cycle/pathway	Abbreviation[a]	mg mL^{-1}	mg in assay[b]	*Vmax* (U mg^{-1})	K_M (mM)	U ml^{-1} assay	Reference
CETCH	Pco	2.5	0.007	12	0.044	0.8	[2]
CETCH	Ccr	6.1	0.001	110	0.17	1	[2]
CETCH	Epi	5.0	0.0005	440	0.08	2	[6]
CETCH	Mcm	4.8	0.001	20	0.14	0.2	[7]
CETCH	Scr	15.1	0.007	29	0.003	2	[13]
CETCH	Ssr	8.3	0.001	3.9	0.013	0.04	[2]
CETCH	Hbs	14.1	0.02	2	0.19	0.4	[8]
CETCH	Hbd	8.8	0.002	26	0.06	0.5	[8]
CETCH	Ecm	8.4	0.002	7	0.06	0.1	[6]
CETCH	Mco	35.0	0.07	0.1	0.03	0.07	[2]
CETCH	Mch	4.5	0.005	1500	n.d.	75	[9]
CETCH	Mcl	5.9	0.025	5	0.01	1	[10]
CETCH	KatE	27.6	0.006	11740	86.5	704	[14]
CETCH	Fdh	27.0	0.03	1.4	0.37	0.4	[4]
CETCH	smPPK2-I	7.5	0.004	12	0.032	0.5	[15]
CETCH	Gor	3.65	0.001	n.d.	n.d.		unpublished
BHAC	BhcA	2.7	0.005	116	0.23	6	[3]
BHAC	BhcD	5.8	0.003	57	0.2	2	[3]
BHAC	BhcB	36.3	0.025	358	0.09	90	[3]
BHAC	BhcC	43.4	0.004	1	2.9	0.04	[3]
BHAC	Mdh	23	0.002	1611	0.04	32	[16]
BHAC	MtkAB	10.2	0.05	n.d.	n.d.		unpublished
Terpene	PhaA	19	0.002	81	0.4	2	[12]
Terpene	Hmgs	10	0.005	1.5	0.01	0.08	[12]
Terpene	Hmgr	3.6	0.03	4	0.02	1.2	[12]
Terpene	Mvk	17.9	0.005	8	0.07	0.4	[12]
Terpene	Pmvk	18.6	0.005	15	0.008	0.8	[12]
Terpene	Mdc	11.8	0.03	4	0.1	1.2	[12]

Terpene	Idi	4.6	0.025	2.1	0.0035	0.5	[17]
Terpene	Gpps	10	0.005	7	0.005	0.4	[12]
Terpene	IspA	9.5	0.005	n.d.	n.d.		
Terpene	LimSyn	34	0.08	n.d.	n.d.		[12]
Terpene	SabSyn	29	0.06	n.d	n.d.		[12]
Terpene	PinSyn	19	0.08	n.d.	n.d.		[12]
Terpene	BisabSyn	29	0.04	n.d.	n.d.		
Terpene	FarSyn	9	0.04	n.d	n.d.		
Terpene	PK/LDH	1U/µl	0.0005	n.d.	n.d.		
PKS	PKS_{SgcE}	20.2	0.03	n.d.	n.d.		
PKS	TE_{SgcE}	15	0.06	n.d.	n.d.		
PKS	Pcc*	3.1	0.03	n.d.	n.d.		

[a] Refer to Table S3 for enzyme name and source [b] Amount corresponds to 100 µl assay volume

Table S7 Synthesized genes

β-Farnesene synthase

GCTAGCATGGCGACTAACGGCGTAGTGATTAGCTGCCTGCGTGAAGTGAGACCGCCGATGACTAAACATGCGCCTAGCATGTGGACTGACACCTTCTCTAAC
TTTAGTCTTGATGATAAAGAGCAACAGAAATGTAGTGAAACCATTGAAGCACTGAAACAGGAAGCGCGCGGTATGCTGATGGCTGCTACCACTCCACTGCAGC
AGATGACCCTGATCGACACCCTGGAACGTCTGGGTCTGTCCTTCCATTTCGAAACCGAAATCGAATATAAAATTGAACTGATCAACGCTGCTGAAGACGACGG
TTTCGACCTGTTTGCGACCGCTCTGCGTTTCCGTCTGCTGCGTCAGCACCAGCGTCATGTTTCTTGTGACGTTTTCGATAAATTCATCGATAAAGATGGTAAAT
TCGAAGAATCTCTGTCTAACAACGTTGGTGGCCTGCTGTCCCTGTACGAAGTTGCGCACGTGGGTTTCCGTGAAGAACGCATCCTGCAGGAAGCTGTGAACT
TCACCCGTCACCACCTGGAAGGTGCTGAACTGGACCAGAGCCCGCTGCTGATCCGTGAAAAAGTTAAACGTGCGCTGGAACACCCGCTGCACCGTGACTTC
CCGATAGTCTACGCACGTCTGTTCATCTCTATTTATGAAAAGGACGACTCGCGCGATGAACTGTTGCTGAAACTCAGTAAGGTGAACTTTAAATTTATGCAGAA
CCTGTATAAAGAAGAACTGTCTCAGCTGTCTCGTTGGTGGAACACCTGGAACCTGAAATCTAAACTGCCGTATGCACGTGATCGTGTTGTTGAAGCATACGTTT
GGGGCGTTGGTTACCACTACGAACCGCAGTACTCCTATGTTCGTATGGGTCTGGCTAAAGGTGTTCTGATCTGCGGTATTATGGATGACACCTATGACAACTA
CGCTACCCTGAACGAAGCACAGCTGTTCACCCAGGTTCTGGATAAATGGGATCGTGACGAAGCGGAACGTCTGCCGGAATACATGAAAATCGTTTACCGTTT
CATCCTGTCTATCTACGAAAACTACGAACGTGATGCTGCGAAACTGGGTAAATCCTTCGCTGCTCCGTACTTCAAAGAAACCGTGAAACAGCTGGCGCGTGCA
TTCAACGAAGAACAGAAATGGGTAATGGAACGTCAGCTGCCGTCCTTCCAGGACTACGTGAAAAACAGTGAAAAAACCTCCTGCATCTACACCATGTTCGCGA
GCATCATCCCAGGCCTGAAATCCGTTACCCAGGAAACCATCGACTGGATCAAATCTGAACCGACCCTGGCAACCTCTACCGCGATGATCGGTCGCTACTGGA
ACGATGCGAGCTCTCAGCTGCGTGAATCTAAAGGCGGTGAAATGCTGACCGCTCTGGACTTCCACATGAAAGAATACGGTCTGACCAAAGAAGAAGCTGCGT
CTAAATTCGAAGGCCTGGTGGAAGAAACTTGGAAAGATATCAACAAAGAATTCATCGCGACCACTAACTACAACGTTGGCCGTGAAATCGCTATCACCTTCCT
GAACTACGCGCGTATCTGCGAAGCAAGCTATAGCAAAACCGACGGTGACGCGTACAGCGATCCGAACGTTGCGAAAGCGAACGTTGTGGCGCTGTTCGTTG
ATGCTATCGTTTTCTAAGTCGAC

α-Bisabolene synthase

CAGTTCCCGCAGACCGTTGATTGGATTCTTAAAAACCAGTTGAAAGATGGCAGCTGGGGCATTCAGTCCCACTTCCTGCTGTCTGACCGTCTCCTGGCCACTC
TGTCTTGCGTGCTGGTTCTGCTGAAATGGAACGTTGGCGACCTGCAAGTTGAACAGGGCATCGAATTCATTAAATCTAACCTGGAACTGGTTAAAGATGAAAC
TGATCAGGATTCTCTTGTAACTGACTTCGAAATCATCTTCCCGTCCCTGCTGCGTGAAGCGCAGTCTCTGCGTCTCGGCCTGCCTTATGACCTGCCGTACATC
CATCTGCTGCAGACTAAACGCCAGGAACGTCTGGCAAAACTGAGCCGTGAAGAAATTTACGCGGTTCCGAGCCCGCTGCTGTACTCTCTGGAAGGTATCCAG
GACATCGTAGAATGGGAACGCATCATGGAAGTTCAATCCCAGGATGGTAGCTTCCTGAGCTCTCCGGCTAGCACTGCATGCGTCTTTATGCACACCGGTGAC
GCTAAATGCCTGGAATTCCTGAACTCCGTAATGATCAAATTTGGTAACTTCGTTCCGTGCCTGTACCCGGTAGATCTCCTGGAACGTCTGCTGATTGTTGACAA
CATCGTGCGCCTGGGTATCTACCGTCATTTTGAAAAAGAAATCAAAGAAGCACTGGACTACGTTTACCGTCACTGGAACGAACGTGGCATCGGCTGGGGCCG
CCTGAATCCGATTGCAGACCTGGAAACCACCGCTCTGGGCTTTCGCCTGCTGCGCCTGCACCGCTACAACGTTTCCCCGGCAATCTTCGACAACTTCAAAGA
TGCTAATGGTAAATTCATCTGCTCCACCGGTCAGTTTAACAAAGATGTAGCGTCCATGCTGAACCTCTACCGCGCTTCCCAGCTGGCGTTTCCGGGTGAAAAC
ATCCTCGATGAAGCTAAATCCTTCGCGACCAAATATCTGCGTGAAGCCTTGGAAAAATCTGAAACCAGCAGCGCTTGGAACAACAAGCAAAACCTGTCCCAGG
AGATCAAATACGCGCTTAAAACTTCCTGGCACGCTTCAGTGCCGCGCGTTGAAGCGAAACGTTACTGCCAGGTTTACCGTCCAGACTATGCACGTATCGCAAA
GTGCGTGTACAAACTGCCGTACGTAAACAACGAAAAATTCCTGGAGCTGGGTAAACTGGATTTCAACATTATTCAGTCAATCCACCAGGAAGAAATGAAAAAC
GTGACCTCTTGGTTCCGTGATTCTGGTCTGCCGTTGTTCACCTTCGCGCGTGAACGTCCTCTGGAATTCTACTTCCTGGTTGCCGCAGGTACCTACGAACCGC
AGTATGCAAAATGTCGTTTCCTGTTCACTAAAGTTGCGTGCCTGCAGACTGTTCTGGACGACATGTATGATACCTACGGCACTCTGGACGAACTGAAACTGTTC
ACTGAGGCTGTGCGTCGTTGGGATCTGTCTTTCACCGAAAACCTGCCGGATTACATGAAACTTTGTTACCAGATCTACTATGACATTGTCCACGAAGTGGCGT
GGGAAGCTGAAAAAGAACAGGGTCGTGAACTCGTTTCGTTCTTCCGTAAAGGTTGGGAAGATTACCTGCTTGGTTATTATGAAGAAGCAGAATGGCTGGCTGC
TGAATACGTGCCGACCCTGGATGAGTACATCAAAAACGGTATCACCTCCATCGGTCAACGCATCCTGCTGCTGAGCGGTGTGCTGATCATGGACGGCCAGTT
GCTGAGCCAGGAAGCACTGGAAAAAGTTGATTACCCAGGTCGTCGTGTACTGACCGAACTGAATTCTCTGATCAGCCGTCTGGCGGATGACACCAAAACTTAT
AAAGCGGAAAAAGCACGTGGTGAACTGGCTTCCTCTATTGAATGCTATATGAAAGATCACCCGGAATGTACCGAAGAAGAGGCACTGGATCACATTTACTCCA
TCCTCGAACCGGCGGTTAAAGAACTGACCCGTGAGTTCCTGAAACCGGATGATGTTCCGTTCGCGTGTAAGAAAATGCTGTTCGAAGAAACTCGTGTGACCAT
GGTTATCTTCAAAGATGGTGACGGTTTCGGTGTTTCTAAACTGGAAGTTAAAGACCACATCAAAGAATGCCTGATCGAACCGCTGCCGCTGTAACTCGAG

PKS$_{SgcE}$

CATATGAGCCGTATCGCTATCGTTGGTGTTGCATGCACCTATCCGGACGCAACCACCCCGCGTGAACTGTGGGAAAACGCAGTAGCAGGCCGTCGTGCCTTT
CGTCGTCTGCCGGACGTGCGTATGCGTCTGGACGATTACTGGAACCCGGACCCGACCGTTCCGGACACCTTCTACGCGCGTAACGCGGCTGTTCTGGAAGG
CTGGGAATTCGATCGTGTAGCTCATCGTATTGCTGGTTCCACCTTCCGTAGCACTGATCTGACCCACTGGCTGGCTCTGGACACCGCGACGCGTGCGCTGGC
TGACGCAGGTTTCCCGGCAGGTGAAGGTCTGCCGACTGAACGTACCGGCGTTGTAGTTGGTAACACCCTGACGGGTGAGTTTAGCCGCGCTAACGGTCTGC
GCCTGCGTTGGCCGTACGTTCGCCGCATCCTGGCTGACGCACTGCAGGAACAGGAATGGGACGACGACCGCCTGGGCGCCTTCCTGCGCGGCGTGGAAGA
AGCCTACAAGAAACCGTTCCCGGCTGTCGACGAAGACACGCTGGCCGGCGGCTTGAGCAACACCATTGCTGGTCGCATCTGTAACCACTTTGACCTGAACG
GTGGCGGCTACACGGTTGATGGCGCGTGCTCCTCTAGCTTGCTGAGCATCACCACCGCGGCTACCTCTCTGCAGTCCGGTGACCTGGACGTCGCAGTGGCG
GGTGGCGTTGATCTGAGCATTGACCCGTTTGAAATCATCGGCTTCGCCAAAACTGGGGCGCTGGCGCGTAAAGAAATGCGTCTGTACGATCGTGGCTCCAAC
GGTTTCTGGCCGGGCGAGGGTTGCGGCATGGTTGTTCTGATGCGTGAAGAAGACGCCGTTGCGTCCGGCCACCGCATCTATGCATCTATCGCGGGTTGGGG
CATTAGCTCTGACGGTCAGGGCGGCATTACTCGCCCGGAAGTATCCGGCTACCAGCTGGCACTGTCCCGCGCTTATGACCGTGCCGGTTTCGGTATTGAAAC
CGTGCCGCTGTTCGAGGGTCATGGTACCGGCACCGCGGTAGGGGACGCAACGGAACTGCGTGCGATCATGAGCGCACGTGCGGCGGCGGATCCGCACGC
GCCGTCTGCTGTGATCACCTCTATCAAAGGCATGATCGGTCACACCAAAGCCGCTGCAGGCATCGCTGGCCTGATTAAGGCTGTAATGGCGCTGGACAGCG
GTGTGCTGCCGCCGGCTATTGGTTGTGTTGATCCGCATGACCTGCTCACTGACGAATCGGCGAACCTGCGTGTTCTGCGTAAGGCGGAAAGCTGGCCGGAA
AACGCGCCGTTGCGTGCGGGCATCACCGCGATGGGTTTCGGCGGTATCAACACCCATGTTGTCCTGGATCGCTCCGACGCCTCCGGTCGTCGTCCGGCGGT
TAACCGTCGTACTACTCTGCTGGCGAACTCTCTCCAGGATTCTGAGCTGCTCCTGCTTGACGGTGAGTCCCCGGCGGCGCTGGCGCGTCGTCTGACCCAGG
TGGCGGATTTCGCCGCACAGGTATCCTATGCGCAGCTGGGTGACCTGGCAGCCACGCTCCAGCGTGAACTGCGTGATCTGCCTCACCGCGCCGCCGTAGTG
GCTACCTCTCCAGAAGATGCGGAACTCCGTCTTCGTGGCCTGGCGGAAACCGCCGGCGGTCGTGCACCTGATGATGGTCCGGTATTCAGTCAAGATGGCCG
CGCGTTCCTGGGTACCGCTGCTGAAGGTGCACGTGTAGGCTTCCTGTTTCCGGGTCAGGGCTCCGGTACCTCCACCGCTGGCGGCGCTCTGGCACGTCGCT
TTACTGAAGCAGCAGAAGTGTATGCACGTGCTGGTTTACCTACTGCAGGTGACATGGTTGCTACCCATGTTGCTCAGCCACGTATCGTTACCGGTTCGACCGC
TGGTTTGCGCGTGCTGGAAGCGCTCGGCATCGAAGCTGATATCGCGCTGGGTCATTCCCTGGGCGAACTGTCTGCGCTGCACTGGGCCGGTGCACTGGATG
AAACTACCCTCCTGGAAGCGGCCCGCACGCGCGGCGCGGCTATGGCGGCACACTCTGCGTCTGGTACCATGGCTTCCCTGACTGCCACTCCAGAGGAAGCT
GTGCGCTTAGTGGAAGGTCTGCCGGTGGTGATCTCGGGCTACAACGGCCCGCGTCAGACCGTAGTAGCCGGGACTGTGGAAGCGGTTGAATCCGTTGGCG
AGCGCGCGGCGGCCGCTGAGATTGCGTTCACCCGTTTAGCGGTTAGCCACGCGTTCCATAGCCCGCTGGTAGCTCCGGCTGCCGAATCCTTTGGTGACTGG
CTCGCGAAAGCACCGCTGGGTGGTCTGGGCCGTCGCGTAGTTTCCACCGTGACGGGCGCTGAACTGGAGCGTGACACAGATCTGGCTAAACTCCTTCGTCA
ACAGATTACCGACCCGGTCTTATTTACCCAGGCGGTTCGTGCGGCTGCCGCGGAAGTAGACCTGTTCGTTGAAGTTGGCCCAGGTCGTGTCCTGAGCGTTCT
GGCTGCAGAAACCGCGGGTAAACCGGCGGTTGCGTTGAATACTGACGATGAATCTCTGCGCGGTCTGCTGCAGGTTGTTGGCGCTGCGTTCGTAATCGGCG
CCCCGATCATTCACGAGCGTCTGTTCAATGATCGCCTGACTCGCCCGTTAGAAGTAGGCAAAGAATTCCTGTTTCTGTCAAGCCCGTGTGAACAGGCGCCGG
AATTTACCCTGCCGGCAGCGGCTCGCGAACCGCTGGTGCAGGAGCATGACGCTCCCACCACCGCTGGCGCTGGTGACACTGCTGAAGAATCTGCCCTGGAC
GTCCTGCGTGCGCTGGTTGCGGAGCGTGCTGAACTGCCGTCTGAGCTGATCGATGAAAATTCCTCCCTGCTGGACGATCTGCACATGTCGTCTATCACTGTT
GGCCAGATTGTTAACCAGACCGCAGTGCGTCTGGGTCTGGCACCGTCCAGCATCCCGACCAACTTCGCTACCGCGACCCTGGCTGAACTGGCGTCCGCGCT
GACTACTTTGGTCGAAACCGGCGCGGATCCGACTGCTGCTCCGGTTGTAACGGGTTCCGCGGCGTGGGCCCGTCCTTTCTCTGTCGATCTGGACGAATTAC
CACTGCCGCCGGCGGTGGCTGATGAAAAGGACGGCACTTGGGAATTGTTTACCTCTGCTGATCACCCGTTCGCTGAAGAAGTTCGTCGTGCTCTGCAGGAC
GCGGCTGTAGGTTCTGGTGTTCTAGTCTGCCTGCCGGCTGGCTGCTCTCCGGACCAGCTGGAACTGGCTTTAGATGGCGCACGTAGCGCACTCGCGGGTTC
TCAGGAAGGCCGTTTCGTGCTGGTTCAGCATGATCGTGGTGCTGCTGGTCTGGCAAAAACTCTGCACCTGGAAGCCCCGCACCTCCGCACTACCGTGGTTCA
CACCCCGGTAGCTGACGGTGCTGCTGACCGTGTTGCCGCGGAAGTGGCGGCGACTACCCACTTTTCTGAAGTTCACCTGGACCGTGATGGTACCCGTCGTG
TTCCGGTGCTGCGTGCGCTGCCGTTCGCACCGGACCGTACTGACCAGGTTCTGGGTCCGGATGACGTTCTGCTGGTTACCGGTGGTGGTAAAGGCATCACT
GCTGAATGTGCTCTGGCAGTTGCTGAACGTACCGGTGCGGCTCTGGCGGTGCTGGGCCGTTCTGATCCGGGCTCTGACCAGGATCTGGCTGCGAACCTGG
GTCGTATGCGTGAGTCCGGTATTCGCGTTGCGTACGCGGCCGCTGATGTGACCGATCCGGTCCGTGTTGCGGGCGCCGTTGCTGAACTGACTGGTGCACTG
GGCAGCGTTACCGCTGTTCTGCACGGCGCAGGTCGTAACGAACCGACCGCGCTGGGTGGCCTGGATATGGCAGCGGTGCGCTCGACTCTGGCACCGAAAG
TTGATGGCCTGCGTCACGTGCTCGACGTTGTAGGTGAACAGAACCTGCGTCTGCTTGTTACCTTCGGTTCTATCATTGGTCGCGCTGGCCTCCGTGGCGAAG
CGCACTACGCTACCGCTAACGAATGGCTGGCAGGCCTGACCGAGGATGTTGCACGTCGTAACCCGGACTGTCGTGCACTGTGCATGGAATGGAGCGTGTGG
TCTGGTGTTGGTATGGGTGAAAAACTGTCCGTAGTTGAATCTTTGTCCCGTGAGGGTATCGTTCCGGTTTCTCCGGATCAGGGTATCGAAATCCTGCTGCGCC
TGATCTCCGACCCGGACGCTCCAGTAGTGACCGTTATCAGCGGTCGTACCGAAGGTATCGGTACTGTTCGTCGTGAGCAGCCGCCGCTCCCGCTGCTGCGC
TTCACCGGTGAACCGCTGGTTCGCTACCACGGTGTTGAACTGGTTACCGAAGCGGAACTGAACGCAGGCACTGATCTGTATCTGACCGACCACATGCTTGAT
GGCAACCTGCTCCTGCCGGCAGTGATTGGTATGGAAGCTATGGTTCAGGTTGGCTCTGCGGTTACTGGCCGTCGTGACGTACCGGTCATTGAAGACGCTCG
CTTCCTGCGTCCGATTGTTGTGCCACCGGGCGGTACCACTCGTATCCGTATCGCCGCCACCGTTACCGGTACCGATCGTGTTGACGTTGCGGTTCACGCCCA
GGACACCGGTTTTGCGGCTGAACACTTCCGCGCTCGTCTGGTATACGGCGGCGCAGCGATCCCGGATGGTGCGCCGGACCAGGTGGGCCCGAAAGTACCG
ACCGCACCGCTGGATCCGGCGACTGATCTGTATGGTGGTGTGCTGTTCCAGGGGGAACGCTTCCAGCGTCTGCGTCGTTTCCATCGTGCTGCGGCACGTCA
CGTGGATGCGGAAGTCGCACTGGACACCGCTAGCGGCTGGTTCGCGGGTTTTCTGCCGGGCACTCTGCTGCTCTCTGATCCGGGTATGCGTGACGCTCTGA
TGCACGGGAACCAGGTTTGCGTGCCGGACGCAACCCTGCTGCCAAGCGGCATCGAACGTCTGTACCCGATGGCGGCTGGCGAAGATCTGCCGGAACTGGT
TCGCTATTGCGCAACTGAACGTCATCGCGACGGCGACACCTACGTGTACGACATCGCGGTTCGTACCCCGGACGGTTCTGTAGTTGAACGTTGGGACGGTCT
GACGCTGCACGCTGTACGTAAAAGCGACGGTTCCGGCCCATGGGTGGCTCCGCTGCTGGGTTCCTACCTGGAACGTACTCTGGAAGAAGTTCTGGGCACCC
ACGTTGATGTTGCAGTGGAGCCGGTTCCGGCTGATAGCGGTGGTAGCGTTGCTGACCGTCGTAAAGCGACCGCCCGTGCAGTTCAGCGTGCGCTGGGTGAA
TCCGTTAAAGTGCGTTATCGCCCGGATGGTCGTCCGGAACTGGACGGCGTTCGTCGTCTGAGCGCGGCGCACGGCCCAGGCGTGACCCTGGGCGTAGTCG
GTACTACCACTGTAGCATGCGACATCGAAGCCGTGACCGCACGTGGCGCTCAGGAATGGGAAGGTCTGCTGGGTGAACACGGTAACCTGGCAGCTCTGGTA
GCGAAAGAAACCGGTGAAACCCCAGATCACGCGGCGACCCGTGTTTGGACGGCGGTTGAATGTCTGAAAAAAGCTGGTCTGCCGGCGGGCGCACCGCTGA
CCCTGGAACCGCAGGTACGCTCCGGTTGGATCGTGCTGACCGCGGGTGGTCTGCGCATCGCCACCTTTGCGACCACCCTGCGTCACGTTGAAGAACCGGTA
GTGCTGGCGTTCCTGACCGCAGGCACTGATGATGCTGCGCCGGGCTCTGCTCGTGCTTAAAAGCTT

TE$_{SgcE}$

CATATGACCGCGACCAACCCGGATTACTTCGAACTGCGTCACACCGTTGGTTTCGAAGAAACCAACCTGGTTGGTAACGTTTACTACGTTAACTACCTGCGTT
GGCAGGGTCGTTGCCGTGAACTGTTCCTGAAAGAACGTGCGCCGTCTGTTCTGGCTGAAGTTCAGGAAGACCTGAAACTGTTCACCCTGAAAGTTGACTGCG
AATTCTTCGCTGAAATCACCGCGTTCGACGAACTGAGCATCCGTATGCGTCTGTCTGAACTGCGTCAGACCCAGCTGGAATTCACCTTCGACTACATCAAACT
GGGTGACGACGGTGGTGAAACCCTGGTTGCGCGTGGTCGTCAGCGTATCGCGTGCATGCGTGGTCCGAACACCGCGACCGTTCCGACCCTGATCCCGGAA
GCGCTGGCGGAAGCTCTGGCGCCGTACTCTGACCGTGCGGGTTCTTACGCGGGTCGTGCGGCTTAAAAGCTT

References

[1] D. M. Peter, B. Vögeli, N. S. Cortina, T. J. Erb, *Molecules* **2016**, *21*, 517.
[2] T. Schwander, L. S. von Borzyskowski, S. Burgener, N. S. Cortina, T. J. Erb, *Science* **2016**, *354*, 900-904.
[3] L. Schada von Borzyskowski, F. Severi, K. Kruger, L. Hermann, A. Gilardet, F. Sippel, B. Pommerenke, P. Claus, N. S. Cortina, T. Glatter, S. Zauner, J. Zarzycki, B. M. Fuchs, E. Bremer, U. G. Maier, R. I. Amann, T. J. Erb, *Nature* **2019**, *575*, 500-504.
[4] M. Kitagawa, T. Ara, M. Arifuzzaman, T. Ioka-Nakamichi, E. Inamoto, H. Toyonaga, H. Mori, *DNA Res.* **2005**, *12*, 291-299.
[5] a) X. Chen, Z. F. Guo, P. M. Lai, K. H. Sze, Z. Guo, *Angew. Chem. Int. Ed.* **2010**, *122*, 8098-8100; b) Q. Liu, K. Wu, Y. Cheng, L. Lu, E. Xiao, Y. Zhang, Z. Deng, T. Liu, *Met. Engg.* **2015**, *28*, 82-90.
[6] T. J. Erb, J. Retey, G. Fuchs, B. E. Alber, *J Biol Chem* **2008**, *283*, 32283-32293.
[7] T. J. Erb, *University of Freiburg* **2009**.
[8] M. Konneke, D. M. Schubert, P. C. Brown, M. Hugler, S. Standfest, T. Schwander, L. Schada von Borzyskowski, T. J. Erb, D. A. Stahl, I. A. Berg, *Proc Natl Acad Sci U S A* **2014**, *111*, 8239-8244.
[9] J. Zarzycki, A. Schlichting, N. Strychalsky, M. Muller, B. E. Alber, G. Fuchs, *J Bacteriol* **2008**, *190*, 1366-1374.
[10] T. J. Erb, L. Frerichs-Revermann, G. Fuchs, B. E. Alber, *J Bacteriol* **2010**, *192*, 1249-1258.
[11] K. Hoelsch, I. Suhrer, M. Heusel, D. Weuster-Botz, *Appl Microbiol Biotechnol* **2013**, *97*, 2473-2481.
[12] T. P. Korman, P. H. Opgenorth, J. U. Bowie, *Nat. Comm.* **2017**, *8*, 1-8.
[13] B. Söhling, G. Gottschalk, *Eur. J. Biochem.* **1993**, *212*, 121-127.
[14] M. S. Sevinc, W. Ens, P. C. Loewen, *Eur J Biochem* **1995**, *230*, 127-132.
[15] B. Nocek, S. Kochinyan, M. Proudfoot, G. Brown, E. Evdokimova, J. Osipiuk, A. M. Edwards, A. Savchenko, A. Joachimiak, A. F. Yakunin, *Proc Natl Acad Sci U S A* **2008**, *105*, 17730-17735.
[16] D. J. Nicholls, J. Miller, M. D. Scawen, A. R. Clarke, J. J. Holbrook, T. Atkinson, C. R. Goward, *Biochem Biophys Res Commun* **1992**, *189*, 1057-1062.
[17] V. Durbecq, G. Sainz, Y. Oudjama, B. Clantin, C. Bompard‐Gilles, C. Tricot, J. Caillet, V. Stalon, L. Droogmans, V. Villeret, *EMBO J.* **2001**, *20*, 1530-1537.
